# Occurrence and Distribution of Phytochemicals in the Leaves of 17 *In vitro* Cultured *Hypericum* spp. Adapted to Outdoor Conditions

**DOI:** 10.3389/fpls.2016.01616

**Published:** 2016-10-27

**Authors:** Andrea Kucharíková, Souvik Kusari, Selahaddin Sezgin, Michael Spiteller, Eva Čellárová

**Affiliations:** ^1^Institute of Biology and Ecology, Department of Genetics, Faculty of Science, Pavol Jozef Šafárik University in KošiceKošice, Slovakia; ^2^Institute of Environmental Research, Department of Chemistry and Chemical Biology, Chair of Environmental Chemistry and Analytical Chemistry, Technical University of DortmundDortmund, Germany

**Keywords:** *Hypericum* spp., matrix-assisted laser desorption/ionization high-resolution mass spectrometry, localization, secondary metabolites, inter-sectional, intra-sectional

## Abstract

A plethora of plants belonging to the genus *Hypericum* have been investigated so far owing to the biological efficacies of pharmacologically important secondary metabolites produced by several *Hypericum* species. However, there is currently a dearth of information about the localization (accumulation) of these compounds in the plants *in situ*. In particular, the biosynthetic and ecological consequence of acclimatization of *in vitro* cultured *Hypericum* spp. to outdoor conditions is not fully known. Herein, we report an application of matrix-assisted laser desorption/ionization high-resolution mass spectrometry (MALDI-HRMS) to reveal the distribution of major naphthodianthrones hypericin, pseudohypericin, protohypericin, and their proposed precursor emodin as well as emodin anthrone, along with the phloroglucinol derivative hyperforin, the flavonoids quercetin, quercitrin, rutin and hyperoside (and/or isoquercitrin), and chlorogenic acid in *Hypericum* leaves. Plants encompassing seventeen *Hypericum* species classified into eleven sections, which were first cultured *in vitro* and later acclimatized to outdoor conditions, were studied. We focused both on the secretory (dark and translucent glands, other types of glands, and glandular-like structures) as well as the non-secretory leaf tissues. We comparatively analyzed and interpreted the occurrence and accumulation of our target compounds in different leaf tissues of the seventeen species to get an intra-sectional as well as inter-sectional perspective. The naphthodianthrones, along with emodin, were present in all species containing the dark glands. In selected species, hypericin and pseudohypericin accumulated not only in the dark glands, but also in translucent glands and non-secretory leaf tissues. Although hyperforin was localized mainly in translucent glands, it was present sporadically in the dark glands in selected species. The flavonoids quercetin, quercitrin, and hyperoside (and/or isoquercitrin) were distributed throughout the leaves. Rutin was present only within sections *Hypericum, Adenosepalum, Ascyreia*, and *Psorophytum*. Our study provides insights into the prospects and challenges of using *in vitro* cultured *Hypericum* plants, further adapted to field conditions, for commercial purposes.

## Introduction

The genus *Hypericum* contains around 490 plant species that are classified into 36 sections (Crockett and Robson, 2011; Hypericum Online, 2016). These epitomize a large pool of natural resource for phytochemical investigation, particularly given the well-investigated biological potential of several representatives of the genus accumulating pharmacologically important secondary metabolites. The majority of morphological and phytochemical studies have so far been realized on one well-known species, *Hypericum perforatum*, commonly known as St. John's wort. A broad spectra of secondary metabolites with diverse biological activities have been found in this species, for example, the naphthodianthrone derivatives hypericin and pseudohypericin, their supposed precursor emodin, and/or emodin anthrone (Bais et al., 2003; Kirakosyan et al., 2004; Zobayed et al., 2006), phloroglucinols hyperforin, adhyperforin and flavonoids quercetin, quercitrin, rutin and hyperoside and/or isoquercitrin, to name a few (Nahrstedt and Butterweck, 1997; Saddiqe et al., 2010; Zorzetto et al., 2015; Hou et al., 2016; Oliveira et al., 2016). Among these compounds, the effectiveness of hypericin as a natural photosensitizer with implications in photodynamic therapy has been rigorously explored (Zhang et al., 2014; Jendželovská et al., 2016), in addition to studies on its antiviral and antimicrobial properties (Kubin et al., 2005; Karioti and Bilia, 2010; Dementavicius et al., 2016). Furthermore, both hyperforin and adhyperforin have been shown to play a role in inhibition of various neurotransmitter receptors, thereby exhibiting antidepressant efficacy (Laakmann et al., 1998; Jat, 2013). Additionally, St. John's wort extracts have been shown to contain a plethora of flavonoids, which further aid the antidepressant potency of the extracts (Butterweck et al., 2000; Saddiqe et al., 2010; Hou et al., 2016).

Numerous studies have been performed in the last decade for qualitative as well as quantitative enumeration of secondary metabolites in plant tissues of *Hypericum* spp. Analytical methods such as thin layer chromatography (TLC) (Ferraz et al., 2002), high-performance liquid chromatography (HPLC) (Ferraz et al., 2002), liquid-chromatography mass spectrometric analyses (LC-MS as well as LC-HRMS) (Tatsis et al., 2007; Kusari et al., 2009), and nuclear magnetic resonance (NMR) (Porzel et al., 2014) have been successfully used to determine several major and minor compounds in *Hypericum* plants. However, there is only sporadic information about the distribution and localization (or accumulation) of the aforementioned metabolites in different tissues of plants belonging to the genus *Hypericum*. For example, Hölscher et al. (2009) examined the localization of various secondary metabolites in the leaves and reproductive organs of *H. perforatum* and *Hypericum reflexum* by using matrix-free UV-laser desorption/ionization (LDI) imaging. Thunig et al. (2011) studied the localization of selected secondary metabolites in the leaves and petals of *H. perforatum* using indirect desorption electrospray ionization mass spectrometry imaging (DESI-MSI) via an imprint on a porous Teflon surface. This work was followed-up by Li et al. (2013) with direct DESI analyses of the leaves and petals of *H. perforatum*. Recently, we employed matrix-assisted laser desorption/ionization-HRMS (MALDI-HRMS) imaging to reveal, in high spatial resolution with limited sample preparation, the distribution, localization, and subtleties of hypericins and structural analogs and proposed precursors in the secretory as well as non-secretory tissues of three wild-growing *Hypericum* species (*H. perforatum, H. olympicum*, and *H. patulum*) (Kusari et al., 2015). More recently, we explored the interspecific variation in localization of hypericins and phloroglucinols as well as some new compounds using indirect DESI-MSI in the leaves of seventeen different *in vitro* cultured *Hypericum* species (Kucharíková et al., 2016). This study provided an extensive range of dataset on the localization of major secondary metabolites within leaves of such a broad spectrum of *Hypericum* species.

**Figure 1:**
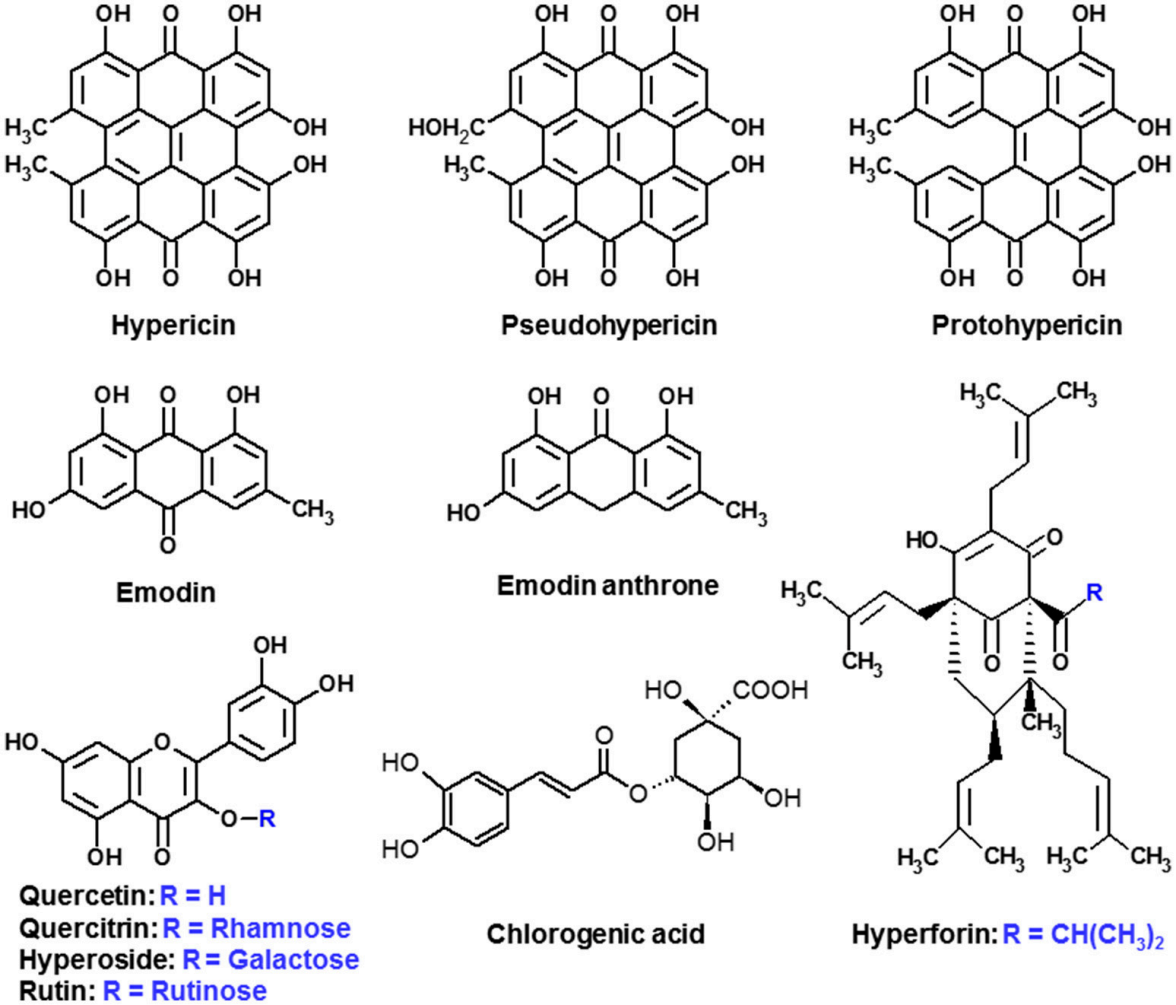
**Chemical structures of the compounds in the present study**.

The present study builds upon and enlarges the aforementioned knowledge and provides a deeper insight to the occurrence and localization (accumulation) of the major compounds (Figure 1) within leaves of the same spectrum of seventeen *Hypericum* species adapted to outdoor conditions using MALDI-HRMS. To the best of our knowledge, this is the first report revealing the occurrence and distribution of important phytochemicals in the leaves of *in vitro* cultured *Hypericum* spp. adapted to outdoor conditions. Notably, our study offers a comparative and qualitative evaluation of the distribution of the target compounds within species cultivated *in vitro* and those adapted to outdoor conditions analyzed by MALDI-HRMS.

## Materials and methods

### Plant material and culture conditions

Experiments were performed with seventeen *Hypericum* species, namely *H. perforatum* L., *H. maculatum* Crantz, *H. androsaemum* L., *H. humifusum* L., *H. bupleuroides* Griseb., *H. kalmianum* L., *H. annulatum* Moris, *H. balearicum* L., *H. tomentosum* L., *H. tetrapterum* Fr., *H. kouytchense* Levl., *H. pulchrum* L., *H. erectum* Thunb., *H. stellatum* N. Robson, *H. monogynum* L., *H. canariense* L. and *H. rumeliacum* Boiss. The plants were vegetatively propagated from *in vitro* cultured seed-derived stock *Hypericum* plants growing on basal Murashige and Skoog medium (Murashige and Skoog, 1962) enriched with Gamborg's B5 vitamins (Gamborg et al., 1968), 30 g L^−1^ sucrose and 2 mg L^−1^ of glycine with pH adjusted to 5.6 before autoclaving. The plants were cultured at 23°C, 34% relative humidity under 16/8 h photoperiod with artificial irradiance of 85 μmol m^−2^ s^−1^.

After acclimation to non-sterile environment in the laboratory, the *Hypericum* plants were adapted to outdoor conditions and consequently grown separately in the pots filled with the soil in area of P.J. Safarik University Botanical garden in Košice, Slovakia. Adapted plants in pots were placed in the air-permeable plastic boxes, watered and transported immediately to INFU, Dortmund for further processing. Fresh leaves of each plant species were isolated and subjected to MALDI-HRMS analyses.

### Reference standards

Hypericin, pseudohypericin, and emodin were obtained from AppliChem (Darmstadt, Germany), hyperforin from Cayman Chemical (USA), quercitrin hydrate (85%) and chlorogenic acid from Sigma-Aldrich Chemie (Steinheim, Germany), quercetin from ABCR GmbH & Co. KG (Karlsruhe, Germany), and hyperoside from Merck KGaA (Darmstadt, Germany). HPLC-grade acetonitrile and HPLC-grade methanol were purchased from J. T.Baker Avantor Performance Materials B.V. (Deventer, Netherlands) and alpha-cyano-4-hydroxycinnamic acid (HCCA) (MALDI grade) from Sigma–Aldrich Life Science (Saint Louis, USA). Demineralized water was distilled two times in-house before use. Reference compounds were not available for protohypericin, emodin anthrone, and isoquercitrin.

### MALDI high-resolution mass spectrometric measurement (MALDI-HRMS)

Samples for MALDI measurement were prepared according to Kusari et al. (2015). Extensive method optimization was also performed according to Kusari et al. (2015). Before starting the measurements with leaves, reference standard (where available) solutions at concentrations of 50 μg mL^−1^ were prepared. 10 μL of each solution was mixed with 10 μL matrix solution. 1 μL volumes of the mixtures were spotted on a stainless steel plate and air dried. Scans were performed on these spots in order to evaluate ionization degree of the compounds in the negative ionization mode. Spray operation was conducted out with the spray device SMALDI Prep (TransMIT GmbH, Giessen, Germany). The matrix solution was sprayed with the parameters as follows: 20 min spray time, matrix flow 15 μL min^−1^, nitrogen gas-stream flow at 4 L min^−1^, and 300 rpm sample platform revolution speed. Prior to sampling, images of leaves were taken from the areas of interest, particularly focusing on dark and further glands (e.g., laminar, ventral) with an optical reflected-light microscope Leica S8AP0 (Leica Microsystems GmbH, Wetzlar, Germany). For MALDI-HRMS measurements, an AP-SMALDI ion source (imagine 10, TransMIT GmbH, Giessen, Germany) was utilized. The beam attenuator was set at 20°. The laser beam was generated via a nitrogen laser (337.1 nm) operating with a pulse frequency of 60 Hz. The source was coupled to a high-resolution mass spectrometer Q Exactive (Thermo Scientific GmbH, Bremen, Germany). All measurements were conducted in the Full-Scan mode within a mass range of *m/z* 100–800 with internal mass calibration with the lock mass *m/z* 333.08808, corresponding to the matrix ion signal [2M−H−CO_2_]^−^ of HCCA in the negative mode. Mass resolution was set at 140,000 at *m/z* 200. Spray voltage was adjusted at 3 kV and maximum injection time at 200 ms.

Different positions of the leaf tissues were morphologically demarcated based on our earlier study (Kusari et al., 2015), with emphasis on secretory and non-secretory structures that could be identified in each *Hypericum* species. Notably, leaf tissue areas containing and lacking dark glands on adaxial and abaxial leaf surfaces of the 17 *Hypericum* species were targeted with laser spots (with laser scan mode: Repetition) of ca. 50 μm diameter in order to obtain intense signals for the analytes. Treatment time was approximately 1–2 min for each position. In case of translucent glands, treatment time was optimized and extended up to 4 min, in order for the laser to penetrate deeper into the tissue. For each type of tissue (e.g., dark gland) on a certain leaf side (e.g., adaxial or abaxial), two shooting positions were randomly chosen and targeted, and the mean intensity values were considered. Data was processed with the software XCalibur (v. 2.2 SP1.48; Thermo Scientific, Bremen, Germany). Mass extraction was performed with deviations ≤ 5 ppm.

## Results

MALDI-HRMS analyses were performed to scrutinize the presence and localization of the important secondary metabolites in the leaves of seventeen selected *Hypericum* species, particularly focusing on the secretory tissues in comparison to the non-secretory tissues. The detailed spatial distribution of naphthodianthrone hypericin, its structural analogs protohypericin and pseudohypericin and potential precursor emodin, emodin anthrone, the phloroglucinol hyperforin, and the flavonoids quercetin, quercitrin, rutin and hyperoside and/or isoquercitrin, along with chlorogenic acid were mapped within fresh, healthy leaves of the seventeen *Hypericum* spp., classified in eleven sections. In addition to the dark and translucent glands, other glandular-like structures were investigated and compared to the surrounding non-secretory tissues, both from the adaxial as well as abaxial side of the leaves. By performing extensive mass spectrometric optimization based on our previous work (Kusari et al., 2015), we aimed to reduce the emergence of interferences and enhance the detection limits for the investigated compounds. Admittedly, due to the lack of a leaf negative control for each species coupled to the complexity of the biological tissues under investigation, low abundant non-continuous signals for compounds that do not have a reference standard, such as emodin anthrone, should be interpreted carefully keeping in mind possible tissue interferences from the leaf surfaces. Notably, low abundant non-continuous signals for emodin anthrone could not be subjected to MALDI-HRMS/MS. Nevertheless, we interpreted these selected data (e.g., for emodin anthrone) with great care, and emphasized on the “trends” while qualitatively working out the distribution pattern in various *Hypericum* spp. Furthermore, the flavonoids hyperoside and isoquercitrin are listed together in the results as it is known that certain *Hypericum* spp. might also contain isoquercitrin (Zorzetto et al., 2015); unfortunately, hyperoside and isoquercitrin could not be distinguished by means of MALDI-HRMS or MALDI-HRMS/MS.

### Spatial chemo-profiling of species belonging to the section *Hypericum*

In the present study, we explored the section *Hypericum*, comprehensively represented by the best studied species of the genus *H. perforatum*, along with three other species, viz. *H. maculatum, H. erectum*, and *H. tetrapterum*. Detailed investigation of different leaves of all four representatives of this section revealed the presence of all our target compounds. Interestingly, hyperforin was found to be localized only in the translucent glands present in the leaves of *H. perforatum*, particularly exemplified by our optimized laser shots on the adaxial surface of the leaf tissues. However, hyperforin could not be detected when investigating the leaves (i.e., translucent glands) from abaxial side. Furthermore, it was remarkable to detect quite intensive signals of hyperforin in the dark glands discerned adaxially on the edges of the leaves. Moreover, low intensity signals were also noticed adaxially in the dark glands located toward the central vein of the leaves (Table 1).

**Table 1:** **Occurrence and accumulation of secondary metabolites in the section ***Hypericum*****.

**Species**	**Area of the leaf**		**Emodin**	**Emodin anthrone**	**Hypericin**	**Pseudohypericin**	**Protohypericin**	**Hyperforin**	**Rutin**	**Hyperoside and/or Isoquercitrin**	**Quercetin**	**Quercitrin**	**Chlorogenic acid**
*H. perforatum sect. Hypericum*	Adaxial	Dark gland; edge	o	−	+	+	−	+	o	−	+	−	+
		Dark gland; inside	o	−	o	−	−	−	−	+	+	−	+
		Translucent gland	o	o	o	−	−	++	o	−	+	o	o
		Non-secretory tissue	o	o	−	−	−	−	−	o	+	−	o
		Reddish mass	o	−	−	−	−	−	−	o	+	−	o
	Abaxial	Dark gland; edge	+	o	+++	+++	++	−	o	+	++	+	o
		Dark gland; inside	+	o	+++	+++	+++	o	−	+	+	+	o
		Translucent gland	+	o	−	o	−	−	−	o	++	o	++
		Non-secretory tissue	o	−	o (n)	+ (n)	−	−	−	−	+	−	o
*H. maculatum sect. Hypericum*	Adaxial	Dark gland; edge	o	−	+	++	−	−	−	o	−	−	+
		Dark gland; inside	o	−	+	++	−	−	−	o	o	−	+
		Translucent gland; edge	o	−	o	+	−	−	−	+	o	−	+
		Translucent gland; inside	o	−	+	++	o	−	−	+	+	o	+
		Non-secretory tissue near middle	−	−	−	−	−	−	−	−	−	−	o
		Non-secretory tissue near dark gland; inside	o	−	−	o	−	−	−	o	o	o	o
	Abaxial	Dark gland; edge	+	−	++	+++	++	−	+	+	+	o	+
		Dark gland; inside	o	−	++	++	++	−	−	+	+	o	o
		Translucent gland; edge	o	−	−	−	−	−	−	−	+	+	+
		Translucent gland; inside	+	o	−	o	−	−	−	−	o	o	o
		Non-secretory tissue; edge	o	−	−	−	−	−	−	−	o	−	+
		Non-secretory tissue near dark gland; inside	o	−	o	+	o	−	−	o	o	o	+
*H. erectum sect. Hypericum*	Adaxial	Dark gland	−	−	+	+	o	−	−	o	o	−	+
		Red gland 1	−	−	−	−	−	−	−	−	−	+	o
		Red gland 2	+	−	−	−	−	−	−	−	−	−	o
		Red gland 3	−	−	−	−	−	−	−	−	−	+	o
		Non-secretory tissue; edge	o	−	−	−	−	−	−	o	o	−	o
	Abaxial	Big dark gland	o	−	+++	+++	++	−	+	+	o	+	+
		Small dark gland	+	−	++	+	−	−	−	−	−	−	o
		Red gland 1; edge	+	o	−	−	−	−	−	+	++	+	+
		Red gland 2; edge	+	+	++(d)	++(d)	−	−	−	++	++	+	+
		Non-secretory tissue; edge	+	−	−	−	−	−	−	−	o	−	o
		Non-secretory tissue; inside	+	o	−	−	−	−	−	−	o	−	o
*H. tetrapterum sect. Hypericum*	Adaxial	Dark gland; edge	+	o	+	+	o	−	−	++	++	+	+
		Translucent gland	o	−	+(n)	+(n)	−	−	−	+	+	+	+
		Reddish dark gland	+	o	++	+++	++	−	−	+	++	+	+
		Unidentified red mass	o	−	o	o	−	−	−	o	++	o	+
		Non-secretory tissue	o	−	−	o (n)	−	−	−	o	+	+	o
	Abaxial	Dark gland; edge	++	+	+++	+++	+++	−	−	+	++	+	o
		Dark gland; inside	++	+	+++	+++	++	−	−	++	++	+	−
		Non-secretory tissue	−	−	−	−	−	−	−	−	−	−	−

*The values are represented as absolute mean signal intensities. +++, > 1E5 (highest); ++, 1E4-9E4; +, 1E3-9E3; o, 5E2-9E2 (lowest); −, not detected; n, non-continuous signal over measurement time; d, detected after long/optimized penetration with laser (> 2.5 min)*.

Naphthodianthrones and the plausible precursor of hypericins, emodin, could be detected in all representatives of the section, both from the adaxial as well as abaxial surface of the leaves, albeit in much higher signal intensities when discerned from the abaxial side of the leaves. Emodin anthrone was detected in some dark and translucent glands in *H. perforatum* in lower intensities than emodin, and only sporadically in the other three plants typically in tissues where emodin was detected. As expected, hypericin and pseudohypericin were localized primarily in the dark glands irrespective of the location of the glands in the leaves. Interestingly, sporadic quantities of hypericin and pseudohypericin could also be identified in some translucent glands and non-secretory tissues throughout the leaves of all the four representatives of the *Hypericum* section. Hypericin and pseudohypercin were also identified in the red glands located at the leaf edges when targeted from the abaxial side of the leaf of *H. erectum* (Supplementary Figures 1, 2). The highest intensities of emodin could be detected in the dark glands of *H. tetrapterum* (Supplementary Figure 3), particularly those on the abaxial side of leaves. It was interesting to note that emodin was present in all types of leaf tissues including the dark glands, translucent glands, morphologically uncharacterized reddish masses (Supplementary Figures 1–4), and even in the non-secretory tissues in the leaves (Table 1). For all the three plants, protohypericin was detected in the dark glands, particularly when discerned from the abaxial surface of the leaves.

All the flavonoids under our study could be detected in the *Hypericum* section. Quercetin, quercitrin, and hyperoside and/or isoquercitrin were present in all four species, while rutin was detected only in the dark glands of *H. maculatum* and *H. erectum*. Further, rutin was found to accumulate in very low quantities in some of the dark and translucent glands in *H. perforatum* (Table 1). Chlorogenic acid was detected in all parts of leaves of plants belonging to all species of this section.

### Spatial chemo-profiling of species belonging to the sections *Drosocarpium, Oligostema*, and *Adenosepalum*

The species *H. rumeliacum* is a key representative of the section *Drosocarpium*, which is known for the presence of hypericin typically localized in numerous bulky dark glands distributed throughout the leaf surface (Kucharíková et al., 2016). In the present study, as anticipated, hypericin, pseudohypericin, and protohypericin were abundantly detected in the dark glands located inside as well as on the edge of the leaves, discerned both from the adaxial and abaxial surfaces. Unlike section *Hypericum*, naphthodianthrones were found to be absent in the non-secretory tissues of *H. rumeliacum* (section *Drosocarpium*). Further, emodin and emodin anthrone were detected solely in the dark glands and not in the surrounding non-secretory leaf tissues; the location of the dark glands in leaves and the surface (adaxial/abaxial) under study did not matter. Hyperforin was absent. Flavonoids, represented by quercetin, quercitrin, and hyperoside and/or isoquercitrin, were found to be present in the dark glands. No flavonoids were found in non-secretory tissues or in the translucent glands, and rutin was not detected in the leaves (Table 2). Chlorogenic acid was also detected in the dark glands.

**Table 2:** **Occurrence and accumulation of secondary metabolites in the sections ***Drosocarpium, Oligostema***, and ***Adenosepalum*****.

**Species**	**Area of the leaf**		**Emodin**	**Emodin anthrone**	**Hypericin**	**Pseudohypericin**	**Protohypericin**	**Hyperforin**	**Rutin**	**Hyperoside and/or Isoquercitrin**	**Quercetin**	**Quercitrin**	**Chlorogenic acid**
*H. rumeliacum sect. Drosocarpium*	Adaxial	Dark gland; edge	+	−	++	+++	+	−	−	o (n)	+	+	−
		Dark gland; inside	+	o	+++	+++	+++	−	o	++	++	++	o
		Non-secretory tissue	−	−	−	−	−	−	−	−	−	−	−
	Abaxial	Dark gland; edge	+	o	+++	+++	+++	−	−	++	++	++	o
		Dark gland; inside	−	−	+	+	−	−	−	−	−	+	−
		Non-secretory tissue	−	−	−	−	−	−	−	−	−	−	−
*H. humifusum sect. Oligostema*	Adaxial	Dark gland; edge	o	o	+	+	−	−	−	o	+	o	+
		Dark gland; inside	−	o	o	o	−	−	−	o	+	−	+
		Translucent gland	o	−	−	−	−	−	−	+	+	+	o
		Non-secretory tissue	o	−	−	−	−	−	−	o	+	o	o
	Abaxial	Dark gland; edge	+	+	+++	+++	+++	−	−	+	++	+	o
		Translucent gland	+	−	−	o (n)	−	−	−	o	+++	o	++
		Non-secretory tissue	o	o	−	o (n)	−	−	−	o (n)	++	o (n)	+
*H. annulatum sect. Adenosepalum*	Adaxial	Dark gland; edge	−	−	o	−	−	−	−	−	o	−	−
		Dark gland; inside	−	−	o	−	−	−	−	−	−	−	−
		Translucent gland	o	−	−	o	−	−	o	o	o	o	−
		Unidentified red spots; edge	−	−	o	o	−	−	−	−	o	+	−
		Non-secretory tissue	−	−	−	−	−	−	−	−	−	−	−
	Abaxial	Dark gland, reddish; edge	+	−	+++	+++	++	−	o	+	++	+	o
		Dark gland; inside	o	−	+	++	o	−	o	−	+	o	−
		Translucent gland	−	−	−	−	−	−	o	−	o	−	−
		Unidentified reddish/orange spot 1; inside	−	−	−	−	−	−	−	−	−	−	−
		Unidentified reddish/orange spot 2; inside	−	−	−	−	−	−	−	−	o	−	−
		Non-secretory tissue	−	−	−	−	−	−	−	−	−	−	−
*H. tomentosum sect. Adenosepalum*	Adaxial	Dark gland; edge	−	−	o	o	−	−	−	−	−	+	−
		Dark gland; inside	−	−	−	−	−	−	−	−	−	−	−
		Translucent gland	−	−	−	−	−	−	−	−	−	−	−
		Non-secretory tissue	−	−	−	o	−	−	−	−	−	−	−
	Abaxial	Dark gland; edge	++	+	+++	+++	+++	−	−	+	++	++	+
		Translucent gland	o	−	−	−	−	−	−	−	+	+	o
		Non-secretory tissue	−	−	−	−	−	−	−	−	o	−	−

*The values are represented as absolute mean signal intensities. +++, > 1E5 (highest); ++, 1E4-9E4; +, 1E3-9E3; o, 5E2-9E2 (lowest); −, not detected; n, non-continuous signal over measurement time; d, detected after long/optimized penetration with laser (> 2.5 min)*.

*H. humifusum* belongs to the section *Oligostema*. Its typical morphology encompasses distribution of small dark glands mostly throughout the leaf margins. Similar to the section *Drosocarpium*, hypericin, pseudohypericin, and protohypericin were found to accumulate in the dark glands, primarily those on the leaf edges as detected from the abaxial side of the leaves. Neither hypericin nor pseudohypericin or protohypericin could be detected in the translucent glands or the non-secretory leaf tissues. Hyperforin could not be detected (< LOD). Emodin and emodin anthrone were detected mainly in the dark and translucent glands discerned from the abaxial side of the leaves. Interestingly however, low amounts of emodin and emodin anthrone could be detected in the non-secretory leaf tissues. Quercetin, the most abundant flavonoid, was detected both in the secretory as well as non-secretory tissues of the leaves, with the highest intensities obtained from translucent glands on abaxial side of the leaves. Hyperoside and/or isoquercitrin and quercitrin were co-distributed mainly in translucent glands on the leaf adaxial surface, as well as in the dark glands on the abaxial surface. Rutin could not be detected (Table 2). Chlorogenic acid was distributed in all parts of leaves.

MALDI-HRMS analyses were also performed on the species *H. annulatum* and *H. tomentosum*, classified under the section *Adenosepalum*. Noticeably, higher signal intensities of the naphthodianthrones under our study could be detected in both species when discerned from the abaxial side of the leaves. As majority of the dark glands in *H. annulatum* are concentrated on the edge of the top-half of the leaf blades, the naphthodianthrones were found to accumulate in highest amounts in these dark glands. Hypericin and pseudohypericin were also present in the morphologically uncharacterized reddish spots (Supplementary Figures 5, 6). Pseudohypericin was found to be absent in the dark glands when measured from the adaxial side of the leaves, plausibly due to the thick cuticle layer.

In *H. tomentosum*, a pair of dark glands are typically present toward the tip of the leaves. Therefore, as expected, hypericin, pseudohypericin, and protohypericin were mainly detected in the dark glands located at the leaf apex. Furthermore, emodin and emodin anthrone were only detected in the marginal dark glands when discerned from the abaxial side of the leaves but not from the adaxial side in both the species under our study. Low intensity signals for emodin, but not emodin anthrone, was also obtained in the translucent glands of *H. tomentosum* from abaxial side of the leaves. However, both emodin and emodin anthrone were found to be absent in the non-secretory tissues of *H. tomentosum* or *H. annulatum* (Table 2). Chlorogenic acid was mainly detected in the dark glands and sporadically in the translucent glands in *H. tomentosum*.

Hyperforin was not detected (< LOD) in the section *Adenospealum*. Flavonoids were found to accumulate typically in the dark glands (abaxial side) of the leaves of both species. According to signal intensities, the most abundant flavonoids were quercetin and quercitrin. Rutin was detected in low abundance only in the dark and translucent glands of *H. annulatum* (Table 2).

### Spatial chemo-profiling of species belonging to the sections *Androsaemum* and *Ascyreia*

In this study, *H. androsaemum* was investigated as a representative species of the section *Androsaemum*. Another three species, namely *H. kouytchense, H. monogynum*, and *H. stellatum* belonging to the section *Ascyreia* were also investigated (Table 3). These four species are known not to contain dark glands on the leaves, and therefore, not to accumulate hypericins. As expected, neither hypericin nor pseudohypericin or protohypericin were found in these four species under our study. Surprisingly, emodin, the proposed precursor of hypericin was found to be present, albeit in low abundance, in all representatives of the section *Ascyreia*. Sporadic non-continuous emodin anthrone signals were detected in the non-secretory tissues in the representatives of the section *Ascyreia*. Emodin and emodin anthrone were not detected in *H. androsaemum*. Moreover, *H. monogynum* did not reveal the presence of emodin during analysis from the adaxial side of the leaves. The remaining three species contained emodin in the translucent glands as well as in the non-secretory tissues throughout the leaves. Additionally, emodin was found to be abundantly localized in the elongated translucent glands on abaxial side of the leaves in *H. stellatum*. Low abundance of emodin was also found in morphologically uncharacterized black tissue masses (Supplementary Figure 7) discerned from the abaxial side of *H. kouytchense* leaves (Table 3). The phloroglucinol, hyperforin, was detected mostly in translucent glands of *H. stellatum*. Chlorogenic acid was detected in all species of the sections *Androsaemum* and *Ascyreia*, even though a pattern of localization could not be ascertained.

**Table 3:** **Occurrence and accumulation of secondary metabolites in the sections ***Androsaemum*** and ***Ascyreia*****.

**Species**	**Area of the leaf**		**Emodin**	**Emodin anthrone**	**Hypericin**	**Pseudohypericin**	**Protohypericin**	**Hyperforin**	**Rutin**	**Hyperoside and/or Isoquercitrin**	**Quercetin**	**Quercitrin**	**Chlorogenic acid**
*H. androsaemum sect. Androsaemum*	Adaxial	Translucent gland; edge	−	−	−	−	−	−	−	−	−	−	o
		Non-secretory tissue; edge	−	−	−	−	−	−	−	−	+	−	o
		Non-secretory tissue; inside	−	−	−	−	−	−	−	o	+	−	o
	Abaxial	Translucent gland; edge	−	−	−	−	−	−	−	−	+	o	o
		Translucent gland; inside	−	−	−	−	−	o (d)	−	−	+	o	o
		Non-secretory tissue; edge	−	−	−	−	−	−	−	−	−	−	−
		Non-secretory tissue; inside	−	−	−	−	−	−	−	−	−	−	−
*H. kouytchense sect. Ascyreia*	Adaxial	Translucent gland; edge	o	−	o	−	−	−	−	+	++	+	o
		Non-secretory tissue; inside	−	−	−	−	−	−	−	−	+	−	−
	Abaxial	Translucent gland; edge	o	−	o	−	−	o (n)	−	o	+	+	o
		Translucent gland; inside	o	−	−	−	−	−	−	+	+	+	−
		Unidentified black masses	o	−	−	−	−	−	−	−	+	+	o
		Non-secretory tissue; edge	−	−	−	−	−	−	−	−	+	+	o
		Non-secretory tissue; inside	o	o (n)	−	−	−	−	−	o	+	+	−
*H. monogynum sect*.	Adaxial	Translucent gland; edge	−	−	−	−	−	−	−	−	o	o	−
*Ascyreia*		Translucent gland; inside	−	−	−	−	−	−	−	−	−	o	−
		Translucent gland; inside	−	−	−	−	−	−	−	−	−	−	−
		Non-secretory tissue; edge	−	o (n)	−	−	−	−	−	−	o	+	−
		Non-secretory tissue; inside	−	−	−	−	−	−	−	−	−	−	−
	Abaxial	Translucent gland; edge	−	−	−	−	−	−	−	−	+	+	−
		Unidentified black dot-like structure	o	−	−	−	−	++	o	o	++	+	o (n)
		Unidentified gland; near the central vein	−	−	−	−	−	o	−	−	−	−	o (n)
		Non-secretory tissue; edge	o	−	−	−	−	−	o	o	o	+	−
*H. stellatum sect*.	Adaxial	Translucent gland; edge	o	−	−	−	−	++	−	−	+	o	o
*Ascyreia*		Translucent gland 1; inside	o	−	−	−	−	++	−	−	+	o	o
		Translucent gland 2; inside	o	o	−	−	−	+ (d)	−	−	o	o	+
		Non-secretory tissue; edge	−	−	−	−	−	−	−	−	−	−	+
		Non-secretory tissue; inside	o	−	−	−	−	−	−	o	o	o	−
	Abaxial	Translucent gland; edge	o	−	−	−	−	++	−	−	−	−	+
		Translucent gland; inside	−	−	−	−	−	−	−	−	o	−	o
		Translucent gland	+	−	o	o	−	++	−	−	+	o	+
		Non-secretory tissue; edge	o	o	−	−	−	−	−	−	−	−	+
		Non-secretory tissue; inside	o	+ (n)	−	−	−	−	−	−	−	−	+

*The values are represented as absolute mean signal intensities. +++, > 1E5 (highest); ++, 1E4-9E4; +, 1E3-9E3; o, 5E2-9E2 (lowest); −, not detected; n, non-continuous signal over measurement time; d, detected after long/optimized penetration with laser (> 2.5 min)*.

The flavonoids quercetin and quercitin were present in all four studied species both in the translucent glands and the non-secretory tissues within the leaves, as well as in the morphologically uncharacterized black leaf tissue masses (Supplementary Figure 7) of *H. kouytchense*. Hyperoside was amply detected only in translucent glands of *H. kouytchense*; however, low abundance of hyperoside was also found in the translucent glands as well as non-secretory leaf tissues of all species under our study (Table 3). Rutin could not be detected.

### Spatial chemo-profiling of species belonging to the sections *Myriandra, Psorophytum*, and *Webbia*

*H. kalmianum, H. balearicum*, and *H. canariense*, belonging to the sections *Myriandra, Psorophytum*, and *Webbia*, respectively, are known to lack dark glands on their leaf blades. Consequently, as anticipated, the naphthodianthrones hypericin, pseudohypericin, and protohypericin as well as emodin could not be detected in these species (Table 4). Interestingly, emodin was sporadically detected in morphologically uncharacterized dark masses spread throughout the leaf cells adjacent to the central vein (Supplementary Figure 8) discerned from the abaxial side of some leaves in *H. canariense*. Strikingly, emodin anthrone could be detected (non-continuous signals) in some glandular as well as non-secretory tissues in *H. balearicum* where emodin was not found (Table 4). The flavonoids quercetin and quercitrin were detected in all four species, discerned both from the adaxial and abaxial sides. Rutin was present in low abundance only in *H. balearicum* (Supplementary Figures 9, 10). Hyperoside and/or isoquercitrin was found to accumulate mainly in *H. kalmianum*, in some specific large translucent glands situated on the edges discerned from the abaxial side of some leaves, as well as in the non-secretory tissues discerned from the adaxial side on the edge of the leaves. Low, discontinuous signals of hyperoside were also detected in *H. balearicum* (Table 4). Chlorogenic acid could not be detected in *H. kalmianum*, even though it was present sporadically in the glandular tissues of *H. balearicum* and in all leaf parts in *H. canariense*.

**Table 4:** **Occurrence and accumulation of secondary metabolites in the sections ***Myriandra, Psorophytum***, and ***Webia*****.

**Table**	**Area of the leaf**		**Emodin**	**Emodin anthrone**	**Hypericin**	**Pseudohypericin**	**Protohypericin**	**Hyperforin**	**Rutin**	**Hyperoside and/or Isoquercitrin**	**Quercetin**	**Quercitrin**	**Chlorogenic acid**
*H. kalmianum sect. Myriandra*	Adaxial	Translucent gland; edge	−	−	−	−	−	−	−	−	o	o	−
		Translucent gland; inside	−	−	−	−	−	−	−	−	o	−	−
		Non-secretory tissue; edge	−	−	−	−	−	−	−	−	−	−	−
		Non-secretory tissue; inside	−	−	−	−	−	−	−	−	+	−	−
	Abaxial	Translucent gland; edge	−	−	−	−	−	−	−	+	+	+	−
		Non-secretory tissue; edge	−	−	−	−	−	−	−	−	−	−	−
		Non-secretory tissue; inside	−	−	−	−	−	−	−	−	−	−	−
*H. balearicum sect. Psorophytum*	Adaxial	Unidentified gland; edge	−	−	−	−	−	−	−	o (n)	−	+	−
		Unidentified gland; inside	−	++ (n)	−	−	−	−	−	−	−	o	o
		Non-secretory tissue; edge	−	−	−	−	−	−	−	−	−	−	−
		Non-secretory tissue; inside	−	+ (n)	−	−	−		−	−	−	+	−
	Abaxial	Unidentified gland; edge	−	−	−	−	−	−	−	o (n)	o	+	−
		Unidentified gland; inside	−	o	−	−	−	−	o	−	+	−	o
		Non-secretory tissue; edge	−	−	−	−	−	−	−	−	−	−	−
		Non-secretory tissue; inside	−	−	−	−	−	−	−	−	−	−	−
*H. canariense sect. Webbia*	Adaxial	Translucent gland; edge	−	−	−	−	−	−	−	−	+	−	o
		Translucent gland; inside	−	−	−	−	−	−	−	o	+	o	+
		Non-secretory tissue; edge	−	−	−	−	−	−	−	+	+	+	+
		Non-secretory tissue; inside	−	−	−	−	−	−	−	−	−	−	o
	Abaxial	Translucent gland; edge	−	−	−	−	−	−	−	−	++	−	o
		Unidentified dark mass; near the central vein	o	−	−	−	−	−	−	−	−	−	+
		Translucent gland	−	−	−	−	−	+	−	−	++	−	o
		Non-secretory tissue; edge	−	−	−	−	−	−	−	−	+	−	+
		Non-secretory tissue; inside	−	−	−	−	−	−	−	−	+	−	+

*The values are represented as absolute mean signal intensities. +++, > 1E5 (highest); ++, 1E4-9E4; +, 1E3-9E3; o, 5E2-9E2 (lowest); −, not detected; n, non-continuous signal over measurement time; d, detected after long/optimized penetration with laser (> 2.5 min)*.

### Spatial chemo-profiling of species belonging to the sections *Bupleuroides* and *Taeniocarpum*

The sections *Bupleuroides* and *Taeniocarpum* are represented by the species *H. bupleuroides* and *H. pulchrum*, both known to produce hypericin exclusively in the reproductive organs. MALDI-HRMS measurements revealed sporadic and scarce occurrence of hypericin, pseudohypericin and emodin in the leaves of *H. bupleuroides* in some morphologically uncharacterized orange or brown spots (Supplementary Figure 11), both from the adaxial as well as abaxial side of the leaves (Table 5). It is compelling that the presence of hypericin in the reproductive organs depends on selected temporal-spatial regulation, which may not be restricted only to the flowering parts of the plants. Both emodin anthrone and protohypericin could not be detected in *H. bupleuroides*. Moreover, emodin was localized in morphologically uncharacterized red, black and brownish spots and masses (Supplementary Figures 12, 13) within the leaves of *H. pulchrum*, whereas emodin anthrone was not detected. Hyperforin and rutin were completely absent in both the species. Quercetin and quercitrin were detected in orange spots (Supplementary Figure 11) in the leaves of *H. bupleuroides*. These two flavonoids were also present in *H. pulchrum*; quercetin as well as hyperoside and/or isoquercitrin could not be detected from the abaxial side of leaves. Low abundance of hyperoside was also found to accumulate intermittently in leaf tissues of *H. bupleuroides* (Table 5). Chlorogenic acid was found in the glandular tissues in *H. bupleuroides* and only sporadically in *H. pulchrum*.

**Table 5:** **Occurrence and accumulation of secondary metabolites in the sections ***Bupleuroides*** and ***Taeniocarpum*****.

**Species**	**Area of the leaf**		**Emodin**	**Emodin anthrone**	**Hypericin**	**Pseudohypericin**	**Protohypericin**	**Hyperforin**	**Rutin**	**Hyperoside and/or Isoquercitrin**	**Quercetin**	**Quercitrin**	**Chlorogenic acid**
*H. bupleuroides sect. Bupleuroides*	Adaxial	Unidentified orange spot	o (n)	−	−	−	−	−	−	−	+	−	−
		Unidentified brown spot; top	o (n)	−	o (n)	−	−	−	−	o	o	o	−
		Unidentified brown spot; bottom	−	−	o (n)	−	−	−	−	o	+	o	−
		Non-secretory tissue	−	−	−	−	−	−	−	−	−	−	−
	Abaxial	Unidentified gland 1	o (n)	−	o (n)	−	−	−	−	−	o	−	o
		Unidentified gland 2	o (n)	−	o (n)	o (n)	−	−	−	o	o	o	o
		Unidentified gland 3	−	−	o (n)	o (n)	−	−	−	−	+	−	o
		Non-secretory tissue	−	−	−	−	−	−	−	−	−	−	−
*H. pulchrum sect. Taeniocarpum*	Adaxial	Translucent gland	o	−	o	o	−	−	−	+	+	o	+
		Unidentified red spot	o	−	o	o	−	−	−	+	+	o	o
		Unidentified red spot; inside	−	−	−	−	−	−	−	o	−	−	−
		Non-secretory tissue	−	−	−	−	−	−	−	o	o	−	−
	Abaxial	Translucent gland	−	−	−	−	−	−	−	−	−	o	−
		Reddish mass around translucent gland	−	−	−	−	−	−	−	−	−	−	o
		Unidentified black dot; inside	−	−	o	o	−	−	−	−	−	o	−
		Unidentified brownish mass; inside	−	−	+	+	−	−	−	−	−	+	−
		Non-secretory tissue	−	−	−	−	−	−	−	−	−	−	−

*The values are represented as absolute mean signal intensities. +++, > 1E5 (highest); ++, 1E4-9E4; +, 1E3-9E3; o, 5E2-9E2 (lowest); −, not detected; n, non-continuous signal over measurement time; d, detected after long/optimized penetration with laser (> 2.5 min)*.

## Discussion

In the present study, we performed extensive MALDI-HRMS analyses to investigate in detail the spatial distribution of the major compounds (Supplementary Figures 14, 15) in the leaves of a broader spectrum of seventeen *Hypericum* spp. classified into eleven different sections. We designed our study to ensure detailed mapping of target compounds in the secretory as well as non-secretory tissues of leaves from both the adaxial and abaxial sides. We analyzed each of the seventeen *in vitro* cultured *Hypericum* spp. adapted to outdoor conditions, section by section, in order to compare our results both from the intra-sectional as well as inter-sectional perspective.

The section *Hypericum*, represented by *H. perforatum, H. maculatum, H. erectum*, and *H. tetrapterum* generally comprises of species accumulating hypericins in specialized dark glands (Robson, 2003), which occur in the aerial parts of the plants (Zobayed et al., 2006). In our present study, hypericin was found to be localized primarily in the dark glands, mainly discerned from the abaxial side of the leaves in all the four representatives of the section, which is consistent with Robson's observation (Robson, 2003). Better detection of hypericins from abaxial side of leaves could be attributed to the absence of a thick epidermis that is present on the adaxial side of leaves, thereby increasing MALDI laser-mediated soft ionization of the compounds. Interestingly, hypericin and pseudohypericin were also detected in low abundance in some of the translucent glands and non-secretory tissues throughout the leaves of all the four species under our study. It is noteworthy that only a few papers dealing with the distribution and localization of secondary metabolites within the genus *Hypericum* have been published so far, and almost all of them are focused typically on *H. perforatum*. The presence of hypericin, pseudohypericin, and protohypericin was also observed in our earlier study in minute amounts around and/or just outside the dark glands in the adaxial side of leaves of *H. perforatum* (Kusari et al., 2015). However, we did not detect these compounds in the translucent glands or in the surrounding non-secretory tissues. Localization of hypericins in the leaves of *H. perforatum* was also realized by Thunig et al. (2011) and Li et al. (2013) using DESI-MSI analyses. Their results confirm the localization of hypericin in the dark glands of leaves and petals, and pseudohypericin and protohypericin in the dark glands of leaves of *H. perforatum*. More recently, we confirmed the localization and/or co-localization of hypericin, pseudohypericin and their protoforms in the dark glands of leaves of the four aforementioned *in vitro* cultured species belonging to the section *Hypericum* using indirect DESI-MSI (Kucharíková et al., 2016).

Emodin, the potential proposed precursor of hypericin (Mazur et al., 1992) was found to be localized in all of the four species under our study from the section *Hypericum*. However, emodin was found to typically accumulate in high abundance in the dark glands of *H. tetrapterum*. In our earlier study (Kusari et al., 2015), emodin was observed to be primarily accumulated in the dark glands, in addition to being dispersed at the vicinity of the glands in the non-secretory tissues, and even throughout other parts of leaves of *H. perforatum*. Our present study corroborates our earlier observation in that emodin is present not only in the dark glands and surrounding non-secretory leaf tissues, but also in morphologically uncharacterized reddish masses (Supplementary Figure 4) and translucent glands within the leaves. In *H. perforatum*, emodin anthrone was detected in some dark and translucent glands in lower intensities than emodin. However, in the other three plants, emodin anthrone could be detected sporadically only in the tissues where emodin was detected.

Within the section *Hypericum*, the phloroglucinol hyperforin could be detected only in *H. perforatum* after longer, optimized treatment of the tissues with laser. Hyperforin was found to accumulate in the translucent glands when discerned from the adaxial side of the leaves. Sporadic occurrence of hyperforin was also observed in the dark glands. Our results are in agreement with the work of Soelberg et al. (2007) who used UV-HPLC to detect hyperforin in the translucent glands as well as in minute amounts in dark glands of *H. perforatum*. In our present study, hyperforin was found to be absent in *H. maculatum, H. erectum*, and *H. tetrapterum*, which is in accordance with our earlier study (Kucharíková et al., 2016). This further demonstrates that the accumulation pattern of selected phytochemicals in these *in vitro* cultured *Hypericum* spp. remain unchanged after acclimatization of the plants to outdoor conditions.

All target flavonoids except rutin were found to be present in the section *Hypericum*. Rutin was detected only in the leaves of *H. perforatum*. Quercetin was present in all selected areas of detection; however, higher incidence of quercetin was observed compared both to its glycoside form (quercitrin) as well as its galactoside form (hyperoside). We made similar observations in our earlier study and the discrimination between quercitrin and hyperoside and/or isoquercitrin was made according to Kusari et al. (2015). Thus, far, it is compelling that higher signal(s) obtained for quercetin can result from ion source fragmentation, thanks to the presence of other minor flavonyl glycosides such as rutin or isoquercitrin. The important biosynthetic intermediate, chlorogenic acid, has been known to be present in several *Hypericum* species since several decades (Seabra and Alves, 1989). In the present study, we could detect chlorogenic acid in all parts of leaves of plants belonging to all species of the section *Hypericum*, corroborating earlier studies reporting its presence in *H. perforatum* (Franklin and Dias, 2011), *H. maculatum* (Kladar et al., 2015), and *H. tetrapterum* (Danova, 2010).

Our next set of plants belonged to the sections *Drosocarpium, Oligostema*, and *Adenosepalum*, all of which include species that demonstrated lack of hyperforin in the leaves. On one hand, absence of hyperforin was also demonstrated by us earlier in the leaves of *in vitro* cultured species *H. rumeliacum* (sect. *Drosocarpium*) and *H. tomentosum* (sect. *Adenosepalum*) (Kucharíková et al., 2016). On the other hand, *H. humifusum* (sect. *Oligostema*) contained traces of hyperforin in translucent glands and *H. annulatum* (sect. *Adenosepalum*) on the upper part of leaf margins (Kucharíková et al., 2016). This reveals that unlike the section *Hypericum*, the accumulation pattern of selected phytochemicals in *in vitro* cultured *Hypericum* spp. belonging to the sections *Oligostema* and *Adenosepalum* are prone to selective variation after acclimatization of the plants to outdoor conditions.

It was interesting to compare the occurrence and accumulation of emodin, the proposed precursor of hypericin, between different sections. In the section *Hypericum*, emodin was detected in the translucent glands and even in the non-secretory tissues in all hypericin-containing species. Strikingly however, in *H. rumeliacum* known to contain higher amounts of hypericin in leaves, emodin was found to localize solely in the dark glands. Localization of emodin anthrone followed a similar pattern as that of emodin, albeit in lower intensities than emodin. Moreover, the sections *Androsaemum, Ascyreia*, and *Myriandra* include species that are all known to lack dark glands on their leaves. Thus far, as expected, we did not detect hypericin, pseudohypericin, or protohypericin in their leaves. Surprisingly, both emodin and emodin anthrone were found to be present in all species of the section *Ascyreia*. These fascinating results open up the following two questions: Is emodin really the precursor of hypericin? What role does emodin and/or emodin anthrone play in *Hypericum* species that lack hypericin?

The last two analyzed species under our study were *H. bupleuroides* (sect. *Bupleuroides*) and *H. pulchrum* (sect. *Taeniocarpum*), which are typically characterized by formation of the dark glands only in reproductive organs. Given this fact, it was remarkable to find even sporadic and/or low abundance of the naphthodianthrones hypericin and pseudohypericin, along with emodin but not emodin anthrone, in some morphologically uncharacterized brownish or reddish spots or masses in the leaves of both species (Supplementary Figures 11–13). This points toward two main possibilities. Firstly, the evolution of the dark glands primarily in the reproductive organs is plausibly to protect the seeds and progenies, given that any tissue-specific regulation of gene expression in the flowers for production of naphthodianthrones should be similar to that in other tissues of the same plant. Secondly, the plausible trafficking of selected bioactive compounds in *H. bupleuroides* and *H. pulchrum* to the non-reproductive organs might be for possible eco-specific plant defense against biotic and abiotic factors. Considering the fact that the plants under our study were acclimatized to outdoor conditions where they are amenable to environmental stressors, trafficking of compounds for the purposes of chemical defense is realistic. The phloroglucinol derivative, hyperforin, was found to be absent in *H. bupleuroides* as well as in *H. pulchrum*, upholding our previous results (Kucharíková et al., 2016). Finally, the flavonoids showed variable distribution within the species without any dark gland in the leaves, further corroborating our previous study (Kusari et al., 2015). Especially, quercetin in both the species and rutin in *H. pulchrum* were detected in aforementioned reddish and brownish spots or masses, indicating possible damage of the leaves and the attempts to quench ROSs that are generated in the process of tissue death.

## Conclusions

The utilization of MALDI-HRMS allowed us to determine the occurrence and localization of several important phytochemicals directly from the leaf surface of seventeen *Hypericum* spp. classified into eleven sections. We were able to simultaneously map the naphthodianthrones hypericin, pseudohypericin, protohypericin, their proposed precursor emodin as well as emodin anthrone, and the phloroglucinol derivative hyperforin along with four flavonoids (quercetin, quercitrin, rutin, and hyperoside and/or isoquercitrin) in the secretory and non-secretory leaf tissues of *in vitro* cultured plants adapted to outdoor conditions. Our broad dataset provides detailed elaboration of plausible biosyntheses and ecological relevance of target compounds in *Hypericum* spp. that have been first cultured *in vitro*, and then adapted to outdoor conditions. On one hand, our study reveals archetypal similarities in the accumulation pattern vis-à-vis ecological function of compounds in selected sections (e.g., *Hypericum*) when comparing *in vitro* cultured plants (Kucharíková et al., 2016) with those adapted later to outdoor conditions (present study). For an important plant genus such as *Hypericum*, particularly containing species such as *H. perforatum* (section *Hypericum*), our results demonstrate the possibilities of using *in vitro* cultured plants further adapted to field conditions for commercial purposes. Certainly, further fundamental research on the feasibility and cost-effectiveness of such an approach should be pursued. On the other hand, our results reveal distinct differences between *in vitro* cultured plants belonging to selected sections (e.g., *Bupleuroides* and *Taeniocarpum*) with those adapted later to outdoor conditions, when comparing the occurrence and localization of our target compounds. In practical terms, this can be expected given the fact that the plants growing outdoor are incessantly frazzled by biotic and/or abiotic environmental factors, which in turn activate trafficking of compounds to the tissue (site) of stress for plant chemical defense responses. Admittedly, the well-known facet of phytochemical variation in different *Hypericum* plant tissues and tissue parts concomitant to morphological, genetic, developmental, population, and environmental factors must be taken into account when evaluating and comparing any data. Nevertheless, the present study exemplifies how MALDI-HRMS can reveal a reliable and robust “pattern” of localization and qualitative abundance of target compounds in selected tissues of various *Hypericum* species.

## Author contributions

SK, MS, and EČ conceived the project, SK and EČ designed the experiments, AK prepared the plants for analyses, SK and SS performed MALDI-HRMS analyses, AK, SK, SS, and EČ analyzed and interpreted the data, AK and SK wrote the manuscript. All co-authors read and approved the manuscript.

## Funding

The present research was supported by the Slovak Research and Development Agency APVV-14-0154 and the Scientific Grant Agency of Slovak Republic VEGA 1/0090/15. SK and MS thankfully acknowledge the Ministry of Innovation, Science, Research and Technology of the State of North Rhine-Westphalia, Germany, and the German Research Foundation (DFG) for granting a high-resolution mass spectrometer and the MALDI imaging high-resolution mass spectrometer.

### Conflict of interest statement

The authors declare that the research was conducted in the absence of any commercial or financial relationships that could be construed as a potential conflict of interest.

## Abbreviations

DESI-MSI: desorption electrospray ionization mass spectrometry imaging
HCCA: α-cyano-4-hydroxycinnamic acid
HPLC: high-performance liquid chromatography
LC-MS: liquid-chromatography mass spectrometry
LDI: laser desorption/ionization imaging
MALDI-HRMS: matrix-assisted laser desorption/ionization high-resolution mass spectrometry
NMR: nuclear magnetic resonance
TLC: thin layer chromatography.
