# Supplementary material for: Occurrence and Distribution of Phytochemicals in the Leaves of 17 In vitro Cultured Hypericum spp. Adapted to Outdoor Conditions
Source: Front Plant Sci. 2016 Oct 27;7:1616. doi: 10.3389/fpls.2016.01616 (PMC5081374; doi:10.3389/fpls.2016.01616)
Supplement: Supplementary file 1 [file Image1.pdf]

*Supplementary Material*

# Occurrence and distribution of phytochemicals in the leaves of 17 *in vitro* cultured *Hypericum* spp. adapted to outdoor conditions

**Andrea Kucharíková[1,†], Souvik Kusari[2,†,*], Selahaddin Sezgin[2], Michael Spiteller[2], Eva Čellárová[1,*]**

*** Correspondence:**
Souvik Kusari
souvik.kusari@tu-dortmund.de

Eva Čellárová
eva.cellarova@upjs.sk

**Table of contents**

**Supplementary Figure 1.** Representative optical image of the abaxial side of *Hypericum erectum* leaf.

**Supplementary Figure 2.** Representative optical image of the adaxial side of *Hypericum erectum* leaf.

**Supplementary Figure 3.** Representative optical image of the adaxial side of *Hypericum tetrapterum* leaf.

**Supplementary Figure 4.** Representative optical image of the adaxial side of *Hypericum perforatum* leaf.

**Supplementary Figure 5.** Representative optical image of the adaxial side of *Hypericum annulatum* leaf.

**Supplementary Figure 6.** Representative optical image of the abaxial side of *Hypericum annulatum* leaf.

**Supplementary Figure 7.** Representative optical image of the abaxial side of *Hypericum kouytchense* leaf.

**Supplementary Figure 8.** Representative optical image of the abaxial side of *Hypericum canariense* leaf.

**Supplementary Figure 9.** Representative optical image of the adaxial side of *Hypericum balearicum* leaf.

**Supplementary Figure 10.** Representative optical image of the abaxial side of *Hypericum balearicum* leaf.

**Supplementary Figure 11.** Representative optical image of the adaxial side of *Hypericum bupleuroides* leaf.

**Supplementary Figure 12.** Representative optical image of the adaxial side of *Hypericum pulchrum* leaf.

**Supplementary Figure 13.** Representative optical image of the abaxial side of *Hypericum pulchrum* leaf.

**Supplementary Figure 14.** Full scan MALDI-HRMS spectra for the target phytochemicals.

**Supplementary Figure 15.** Full scan MALDI-HRMS spectra for the target phytochemicals.

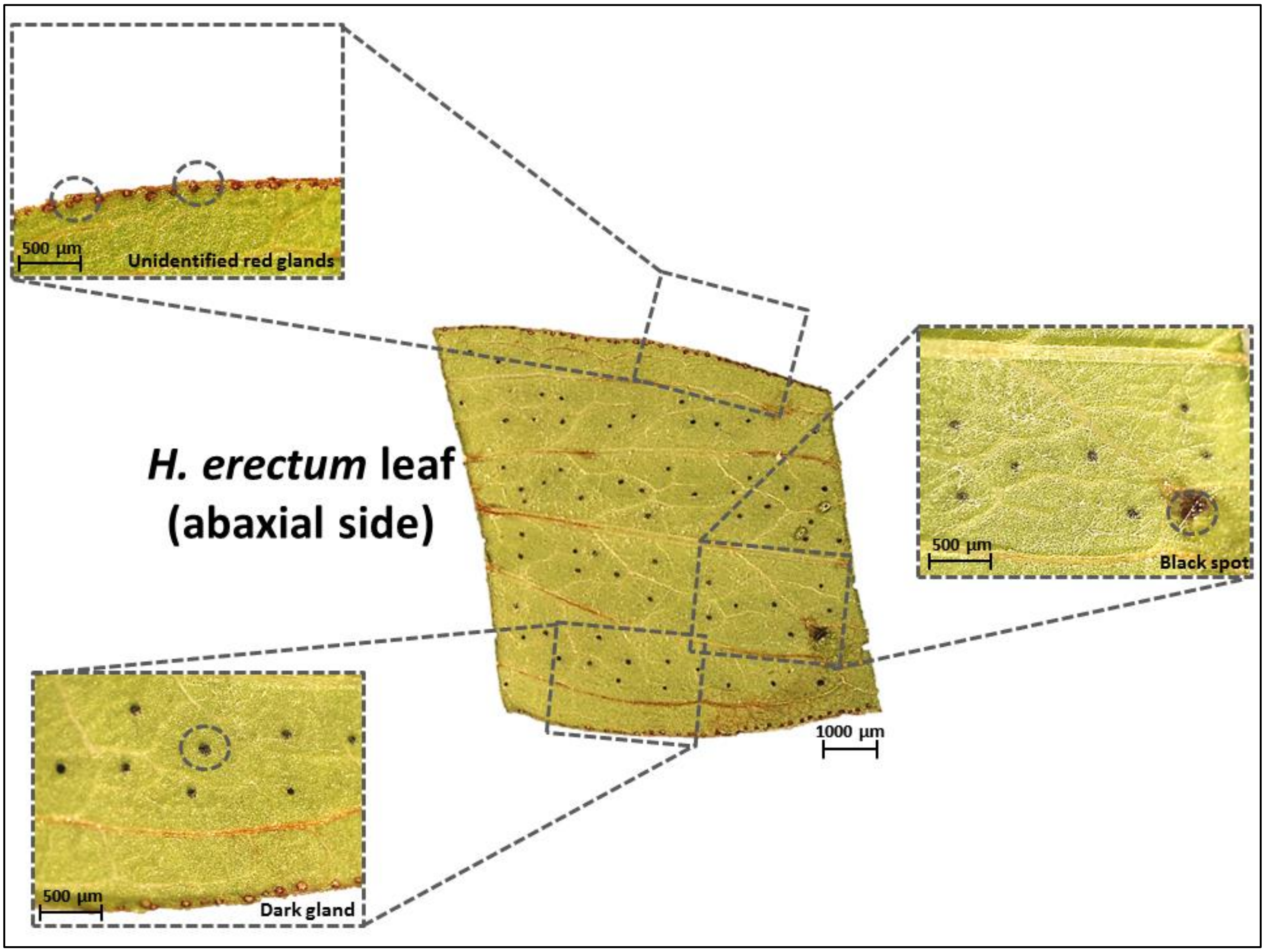


**Supplementary Figure 1.** Representative optical image of the abaxial side of *Hypericum erectum* leaf.

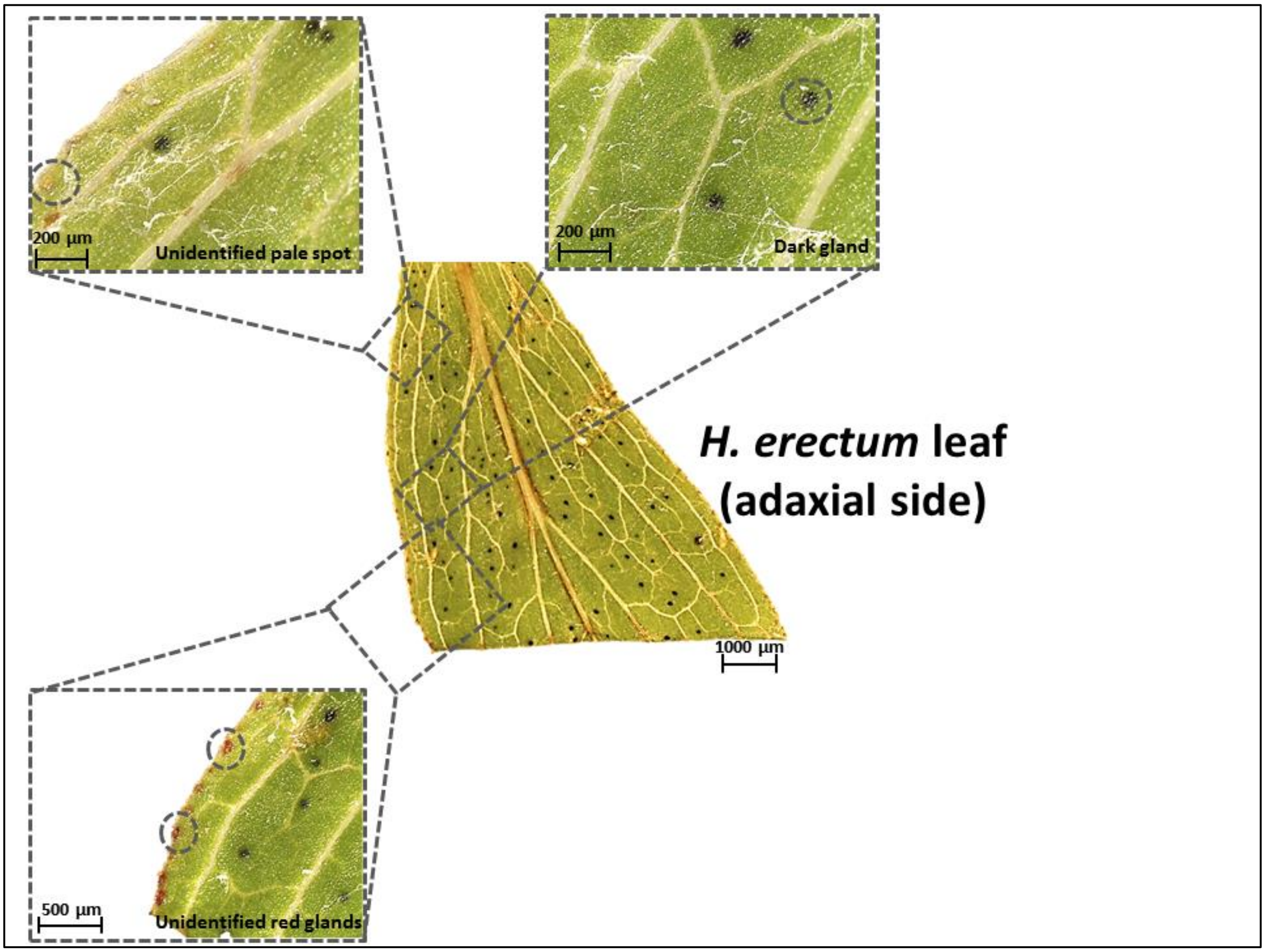


**Supplementary Figure 2.** Representative optical image of the adaxial side of *Hypericum erectum* leaf.

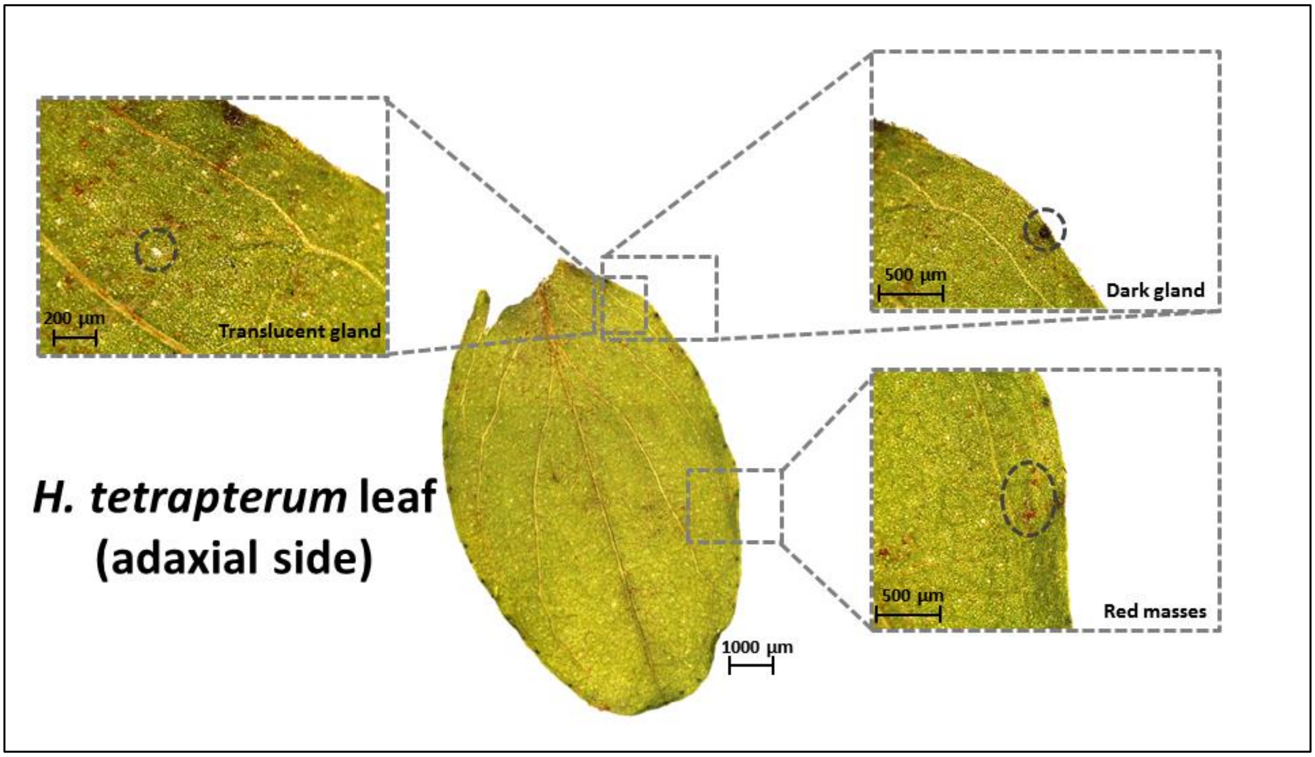


**Supplementary Figure 3.** Representative optical image of the adaxial side of *Hypericum tetrapterum* leaf.

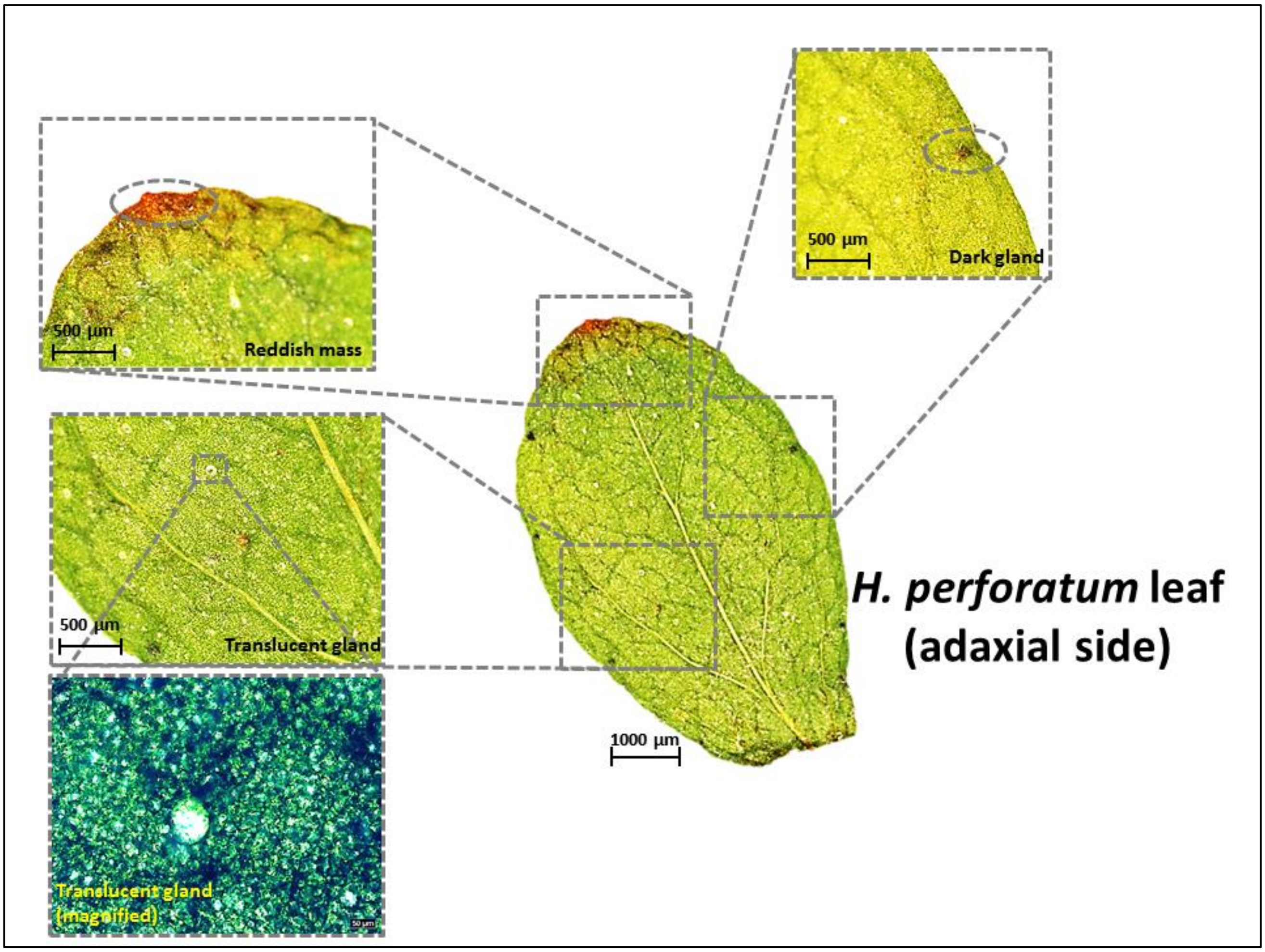


**Supplementary Figure 4.** Representative optical image of the adaxial side of *Hypericum perforatum* leaf.

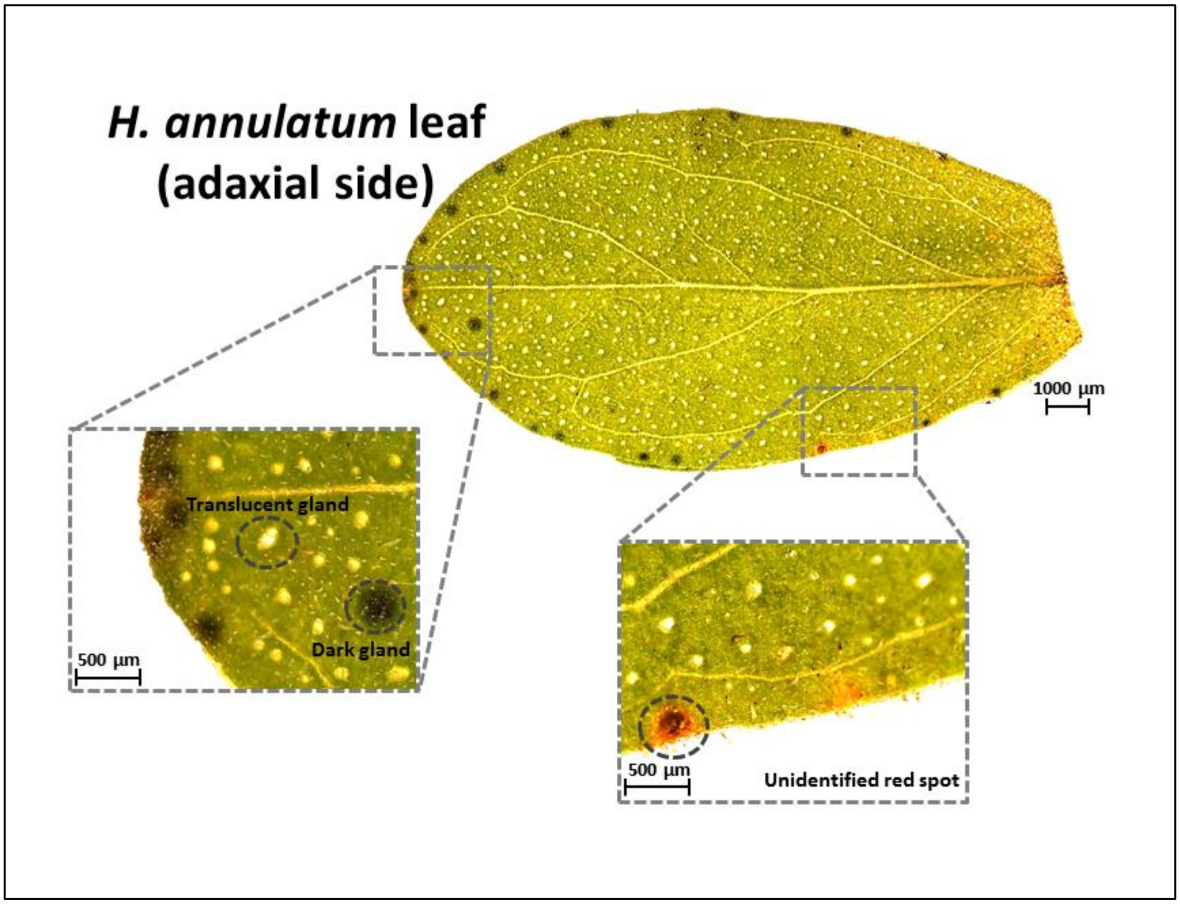


**Supplementary Figure 5.** Representative optical image of the adaxial side of *Hypericum annulatum* leaf.

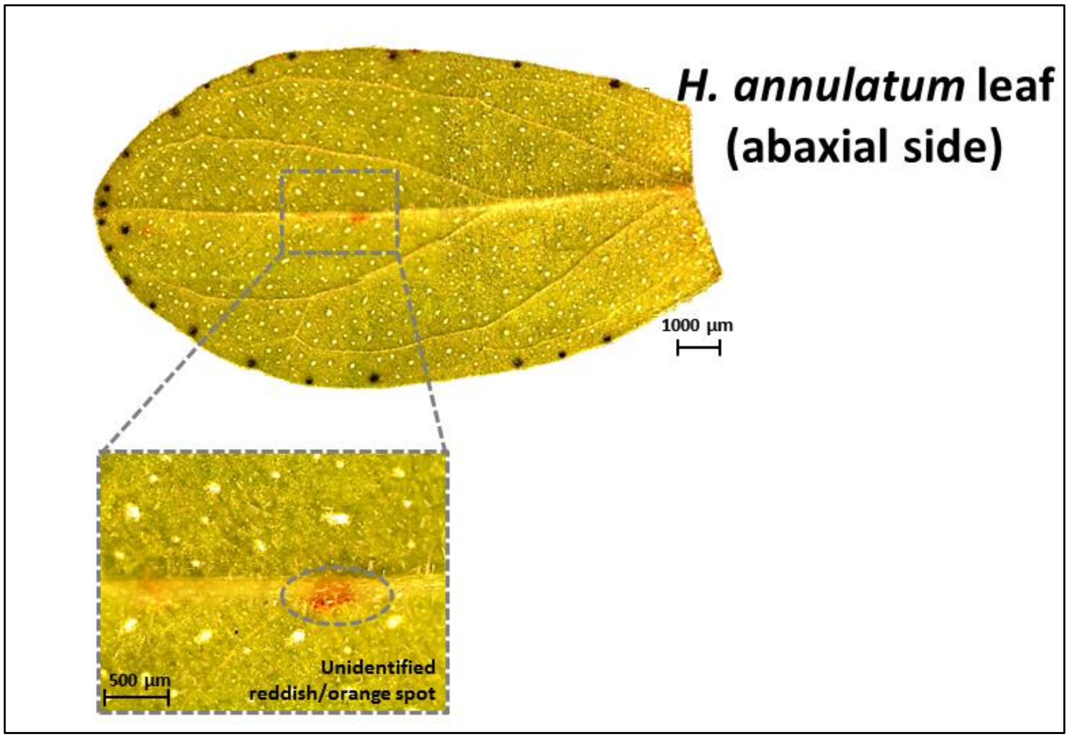


**Supplementary Figure 6.** Representative optical image of the abaxial side of *Hypericum annulatum* leaf.

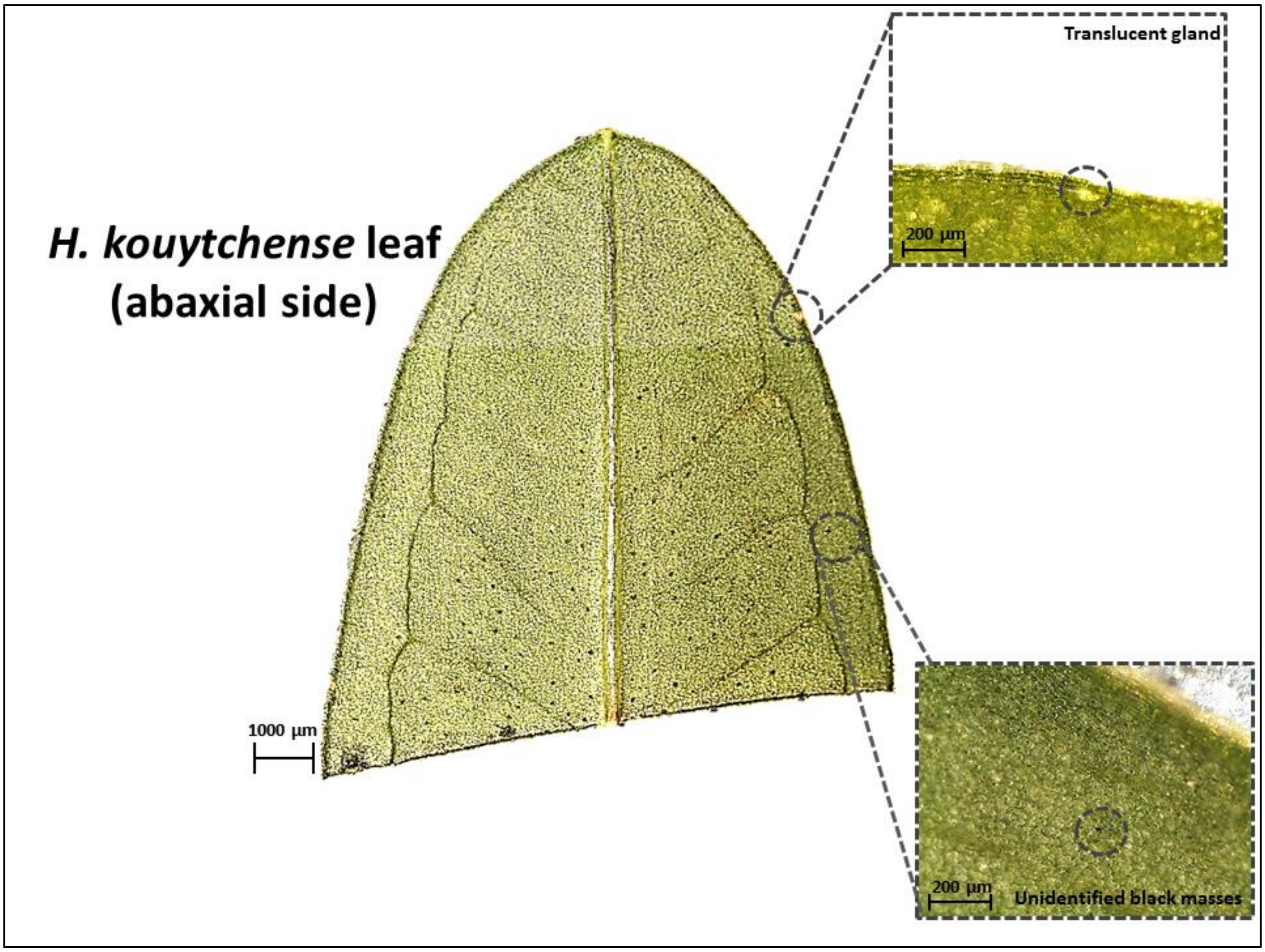


**Supplementary Figure 7.** Representative optical image of the abaxial side of *Hypericum kouytchense* leaf.

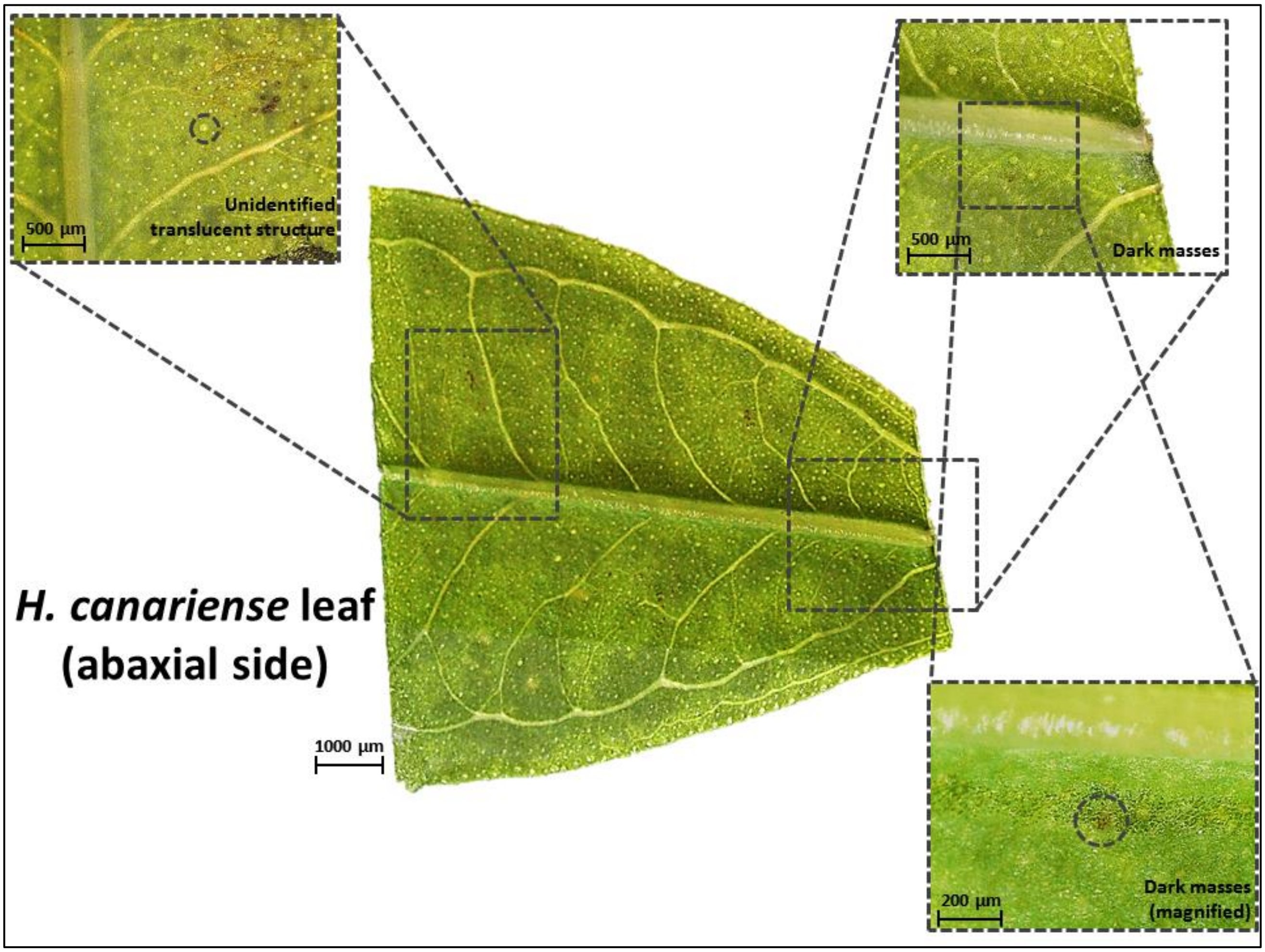


**Supplementary Figure 8.** Representative optical image of the abaxial side of *Hypericum canariense* leaf.

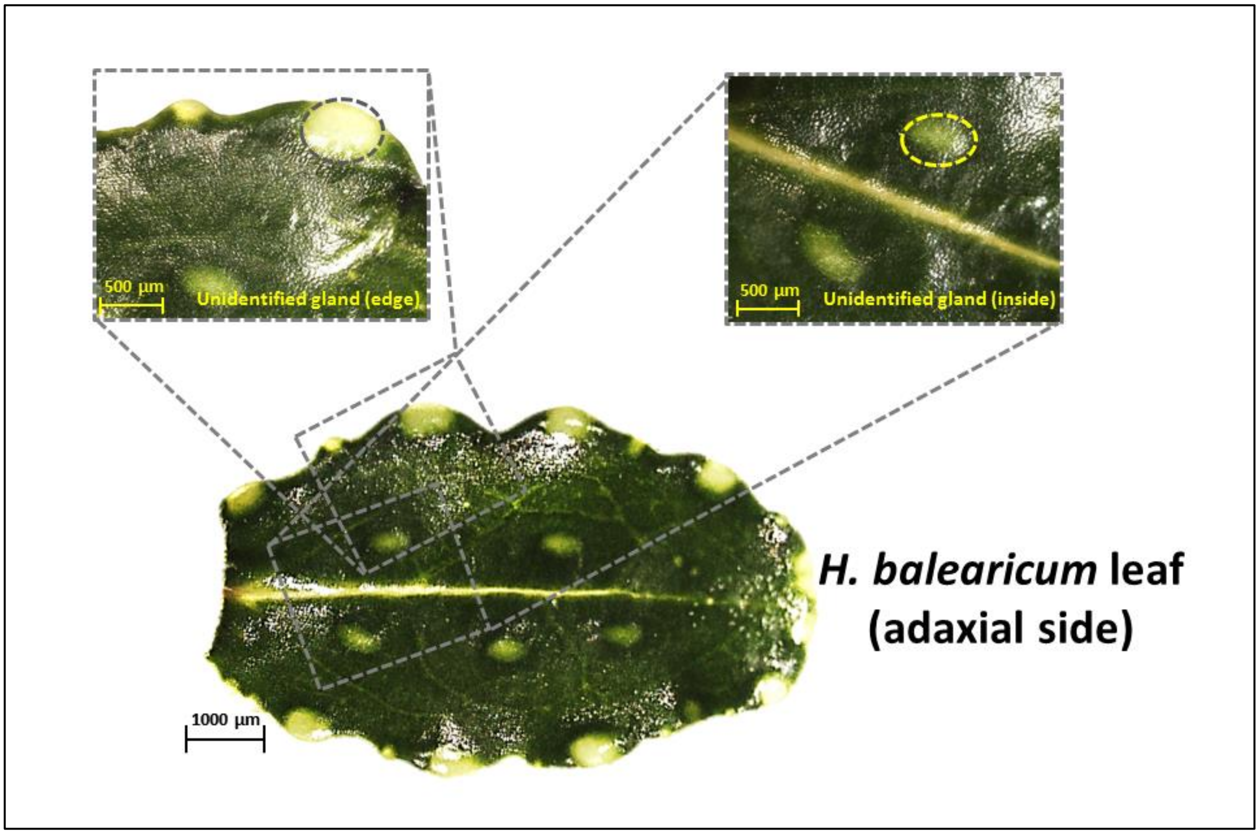


**Supplementary Figure 9.** Representative optical image of the adaxial side of *Hypericum balearicum* leaf.

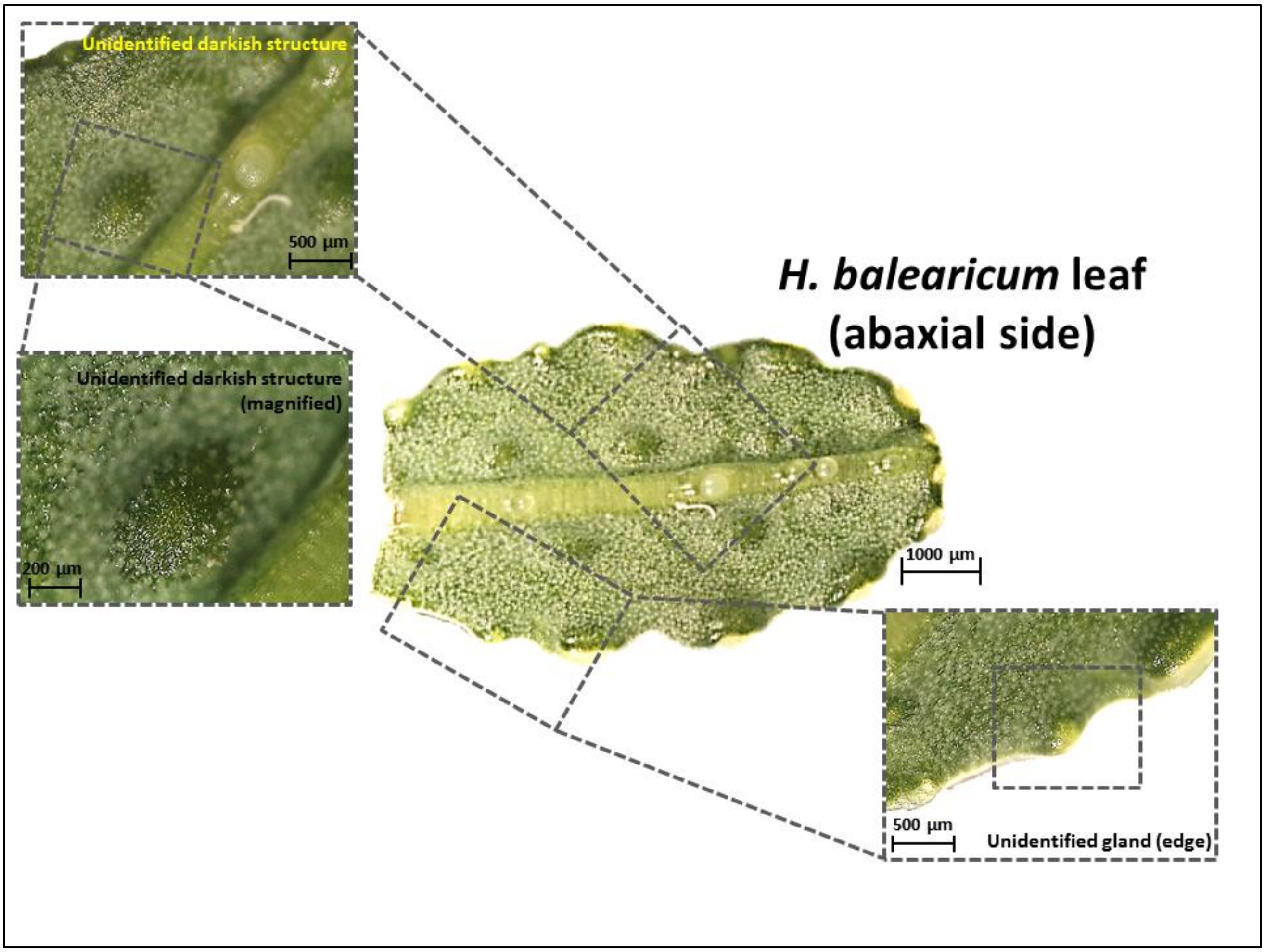


**Supplementary Figure 10.** Representative optical image of the abaxial side of *Hypericum balearicum* leaf.

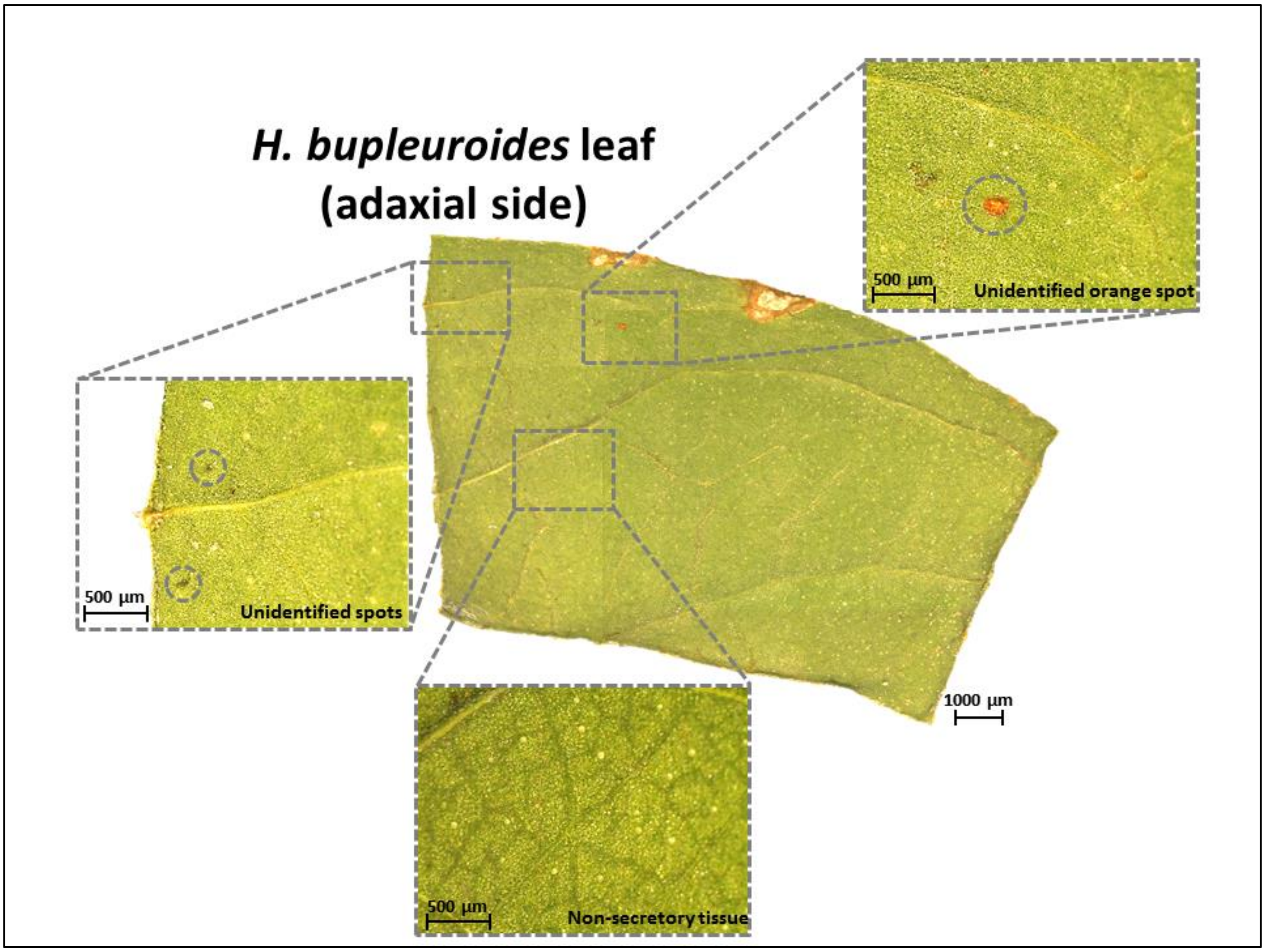


**Supplementary Figure 11.** Representative optical image of the adaxial side of *Hypericum bupleuroides* leaf.

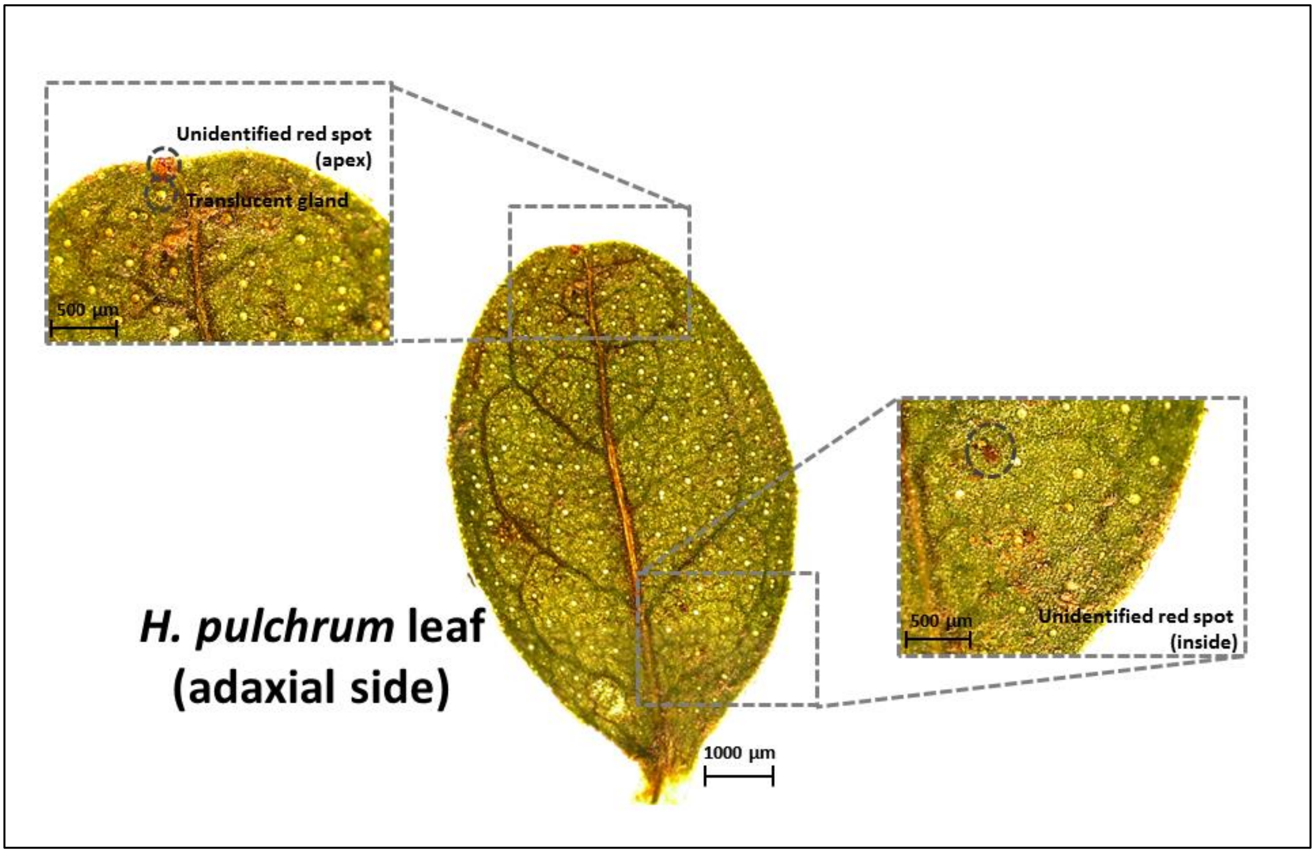


**Supplementary Figure 12.** Representative optical image of the adaxial side of *Hypericum pulchrum* leaf.

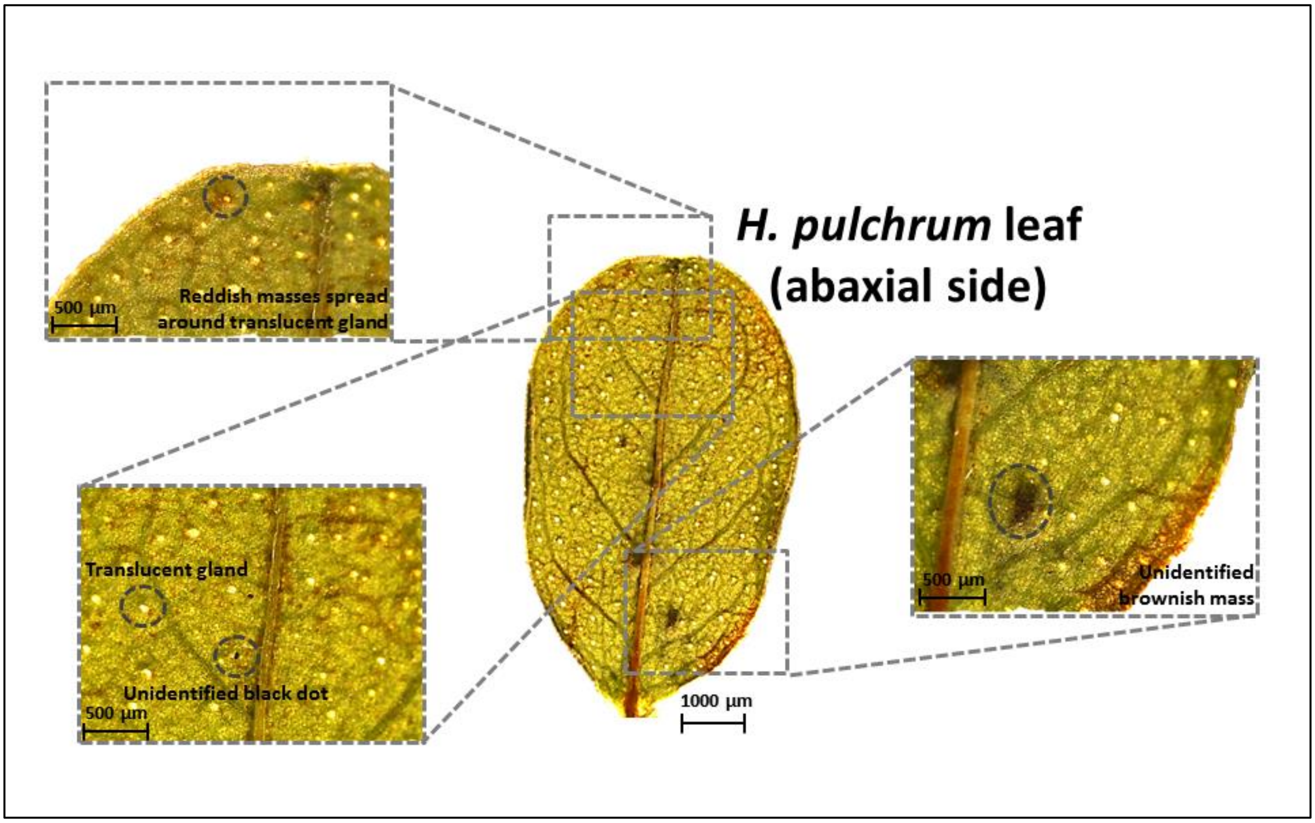


**Supplementary Figure 13.** Representative optical image of the abaxial side of *Hypericum pulchrum* leaf.

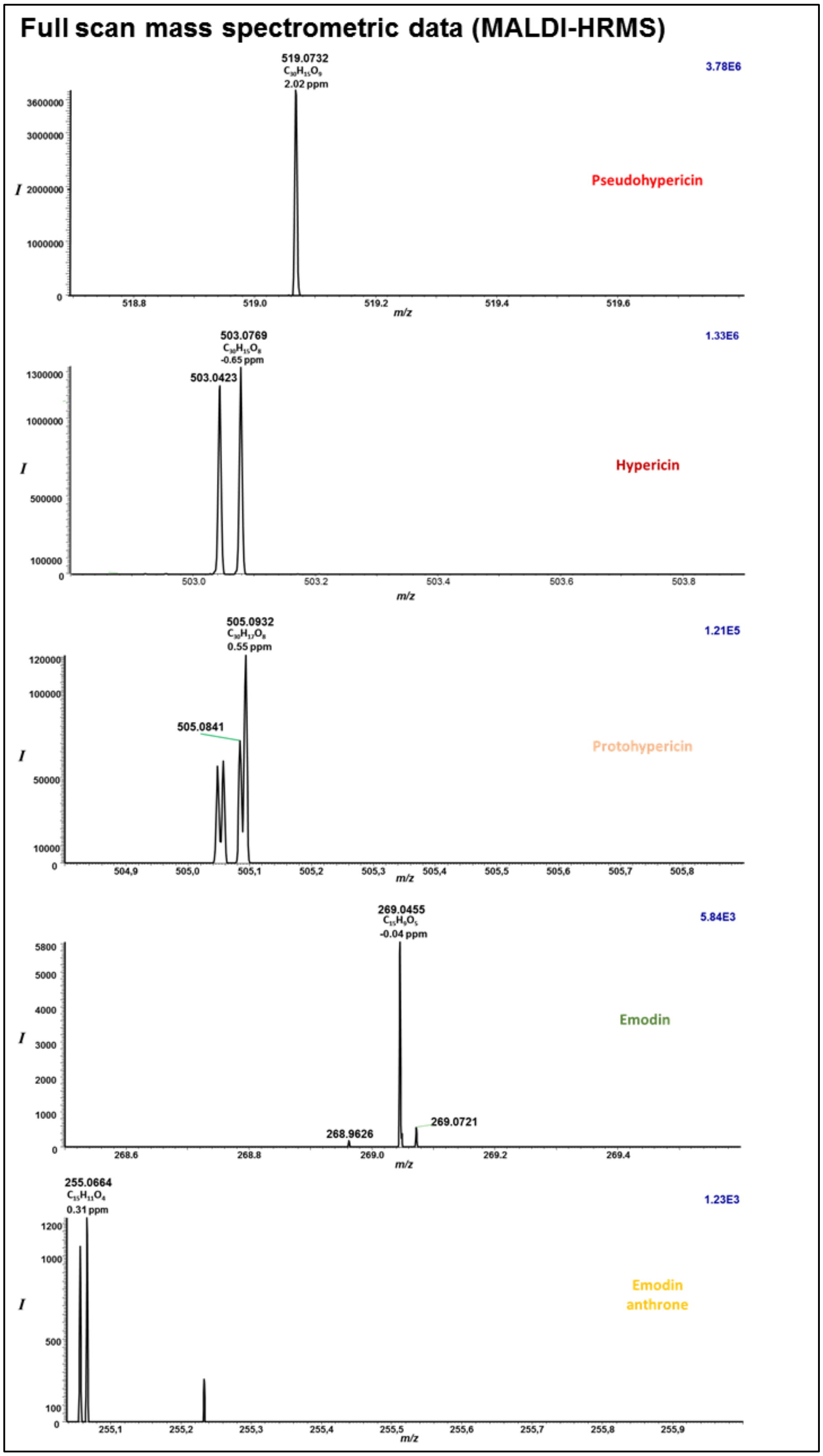


**Supplementary Figure 14.** Full scan MALDI-HRMS spectra for the target phytochemicals.

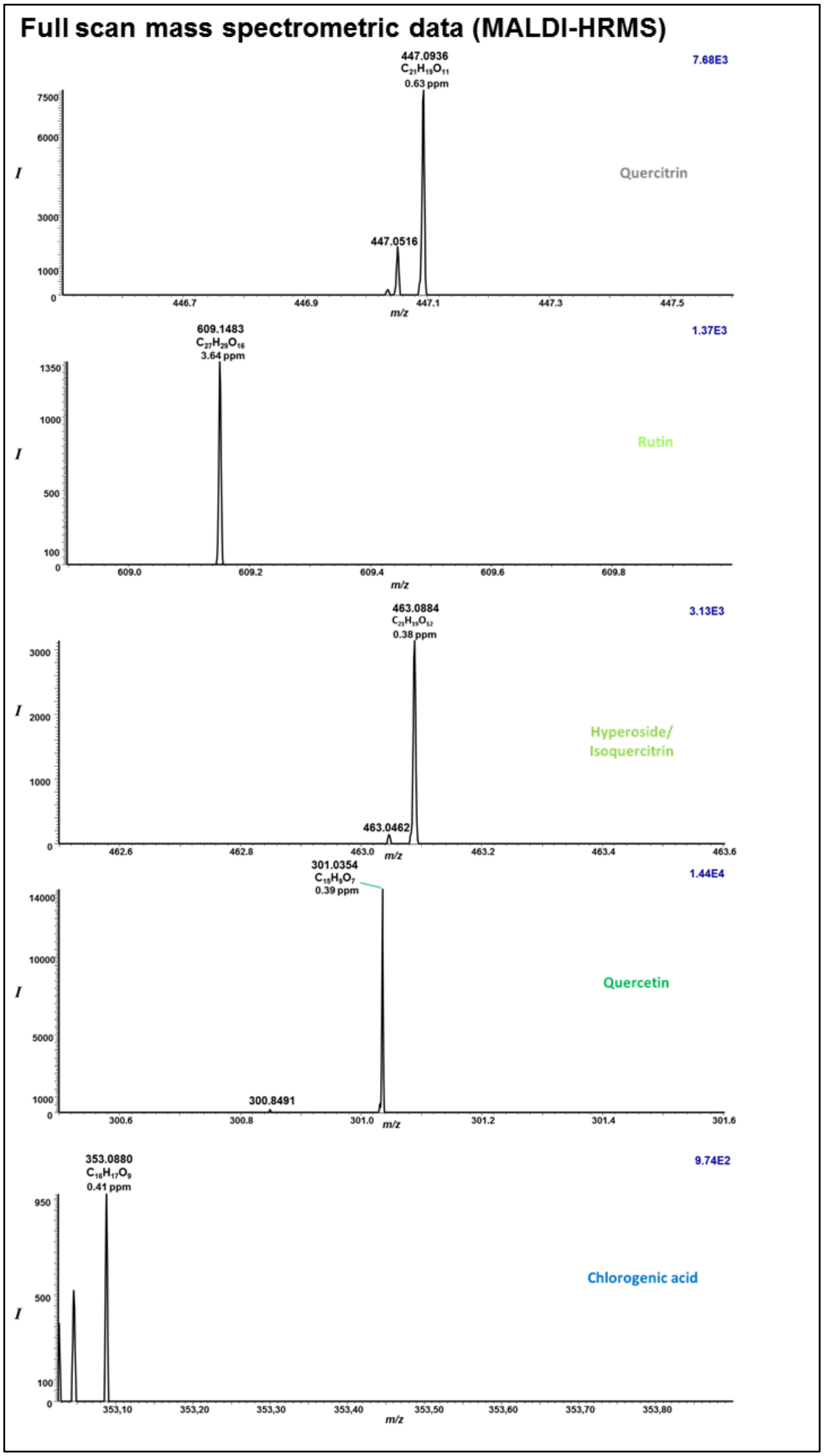


**Supplementary Figure 15.** Full scan MALDI-HRMS spectra for the target phytochemicals.
